# Prevention of high body mass index and eating disorders: a systematic review and meta-analysis

**DOI:** 10.1007/s40519-022-01458-8

**Published:** 2022-08-27

**Authors:** Long Khanh-Dao Le, Eng Joo Tan, Joahna Kevin Perez, Oxana Chiotelis, Phillipa Hay, Jaithri Ananthapavan, Yong Yi Lee, Cathrine Mihalopoulos

**Affiliations:** 1 Health Economics Division, School of Public Health and Preventive Medicine, Monash University, Melbourne, Australia; 2 Deakin Health Economics, School of Health and Social Development, Institute for Health Transformation, Deakin University, Burwood, VIC 3125 Australia; 3 School of Medicine, Translational Health Research Institute (THRI), Western Sydney University, Locked Bag 1797, Penrith, NSW 2751 Australia; 4 Camden and Campbelltown Hospital, SWSLHD, Campbelltown, NSW 2560 Australia; 5 Global Obesity Centre, School of Health and Social Development, Institute for Health Transformation, Deakin University, Burwood, VIC 3125 Australia; 6 School of Public Health, The University of Queensland, Herston, QLD 4006 Australia; 7 Policy and Epidemiology Group, Queensland Centre for Mental Health Research, Wacol, QLD 4076 Australia

**Keywords:** Prevention, Eating disorders, High BMI, Obesity

## Abstract

**Background:**

Eating disorders (EDs) and high body mass index (BMI) are two important public health issues with significant health and cost impacts. The aim of this systematic review and meta-analysis was to establish whether interventions are effective in preventing both issues.

**Methods:**

Electronic databases were searched up to 10 May 2021. Studies were included if they were randomised or quasi-randomised controlled trials that evaluated a preventive intervention (regardless of its aim to prevent ED, high BMI or both) and reported both EDs and BMI-related outcomes. Both narrative synthesis and meta-analysis were used to synthesise the results. Publication bias was also investigated.

**Results:**

Fifty-four studies were included for analysis. The primary aim of the studies was ED prevention (*n* = 23), high BMI prevention (*n* = 21) and both ED and high BMI prevention (*n* = 10). Meta-analysis results indicated that preventive interventions had a significant effect on several ED outcomes including dieting, shape and weight concerns, body dissatisfaction, negative affect, eating disorder symptoms and internalization, with effect sizes ranging from – 0.16 (95% CI – 0.27, – 0.06) to – 0.61 (95% CI – 0.29, – 0.04). Despite several studies that demonstrated positive impacts on BMI, there was no significant effect on BMI-related measures in the meta-analysis. The risk of publication bias was low for the majority of the pooled effect results.

**Conclusion:**

Preventive interventions were effective for either high BMI or EDs. However, there is limited evidence to show that current preventive interventions were effective in reducing *both* outcomes. Further research is necessary to explore the risk factors that are shared by these weight-related disorders as well as effective prevention interventions.

**Level of evidence:**

Level I: systematic review.

**Supplementary Information:**

The online version contains supplementary material available at 10.1007/s40519-022-01458-8.

## Introduction

Eating disorders (EDs) and high body mass index (BMI), defined as BMI greater than 25 kg/m^2^ (or classified as overweight and obesity), are two important public health issues with significant health and cost impacts [1–3]. The Global Burden of Disease study showed that high BMI is amongst the top five risk factors for morbidity and mortality [4] while EDs accounted for 6.6 million (95% CI 3.8–10.6) disability-adjusted life years, and ranked 73^rd^ out of 169 risk factors in 2019. Importantly, EDs are associated with the highest mortality impacts out of all the mental disorders [5]. In younger populations, both EDs and high BMI are among the top three most common chronic conditions in female adolescents while the global prevalence of obesity for male children and adolescents has increased by 7% since 1975 [6].

While interventions for EDs and high BMI are typically developed and implemented independently of each other [7], recent studies have suggested that they share similar and malleable risk factors, which include dieting, media use, body image dissatisfaction and weight-related teasing [8, 9]. In addition, there may be instances where these two disorders are not mutually exclusive, can be present within an individual at the same time and or be present at different periods in a person’s lifetime. [10].

Obesity is a common co-morbidity of EDs, particularly for bulimia nervosa (BN) and binge eating disorder (BED) [11]. For example, according to the WHO World Mental Health Survey, individuals with BN and BED reported significantly higher BMI compared to individuals without a history of ED—approximately 30.7% of individuals with lifetime BED experienced overweight and 32.8% suffered from obesity (Kessler et al. 2013). Similarly, a study of individuals with current EDs reported that 87% of individuals with BED and 33% of individuals with BN had obesity at some point in their lives (Villarejo et al. 2012). Personal and familial history of obesity is also a risk factor for EDs, while restrictive dieting and unhealthy weight control behaviours can increase the likelihood of high BMI [12, 13]. For example, the use of diet pills, laxatives and vomiting were found to be more prevalent in adolescents with overweight compared to their peers with healthy weight [14]. Further, in a case-control study, adult individuals with bulimia were three times more likely to have experienced childhood obesity compared to their healthy counterparts in the control group [15].

Despite shared risk and presentation, it is not known whether there is a single intervention that can simultaneously prevent both high BMI and EDs. High BMI prevention and ED prevention share similar core principles of promoting healthy diets and physical activity [16]. Furthermore, an integrated approach to the prevention of obesity and EDs may be able to reduce risk of unintended consequences such as increasing the risk of eating disorders while aiming to prevent high BMI, and vice versa [7, 8]. For example, preventive interventions for high BMI by monitoring intake and portion control might promote negative shape and weight concerns. Recently, a review found that exposure to both mass media and anti-obesity public health messages can have unintentional effects by exacerbating thin internalization and promoting the thin-ideal [17]. While maintaining a lower or healthy weight is considered a benefit of obesity prevention, it is problematic for prevention of EDs that promotes self-acceptance at any weight and discourages weight-loss [18]. There is growing interest in an integrated approach that focuses on shared risks (e.g., low self-esteem, body dissatisfaction) and protective factors (e.g., healthy eating, regular exercise) for the prevention of obesity and EDs [19].

There is limited evidence on whether there are effective interventions that can provide simultaneously prevent both high BMI and EDs. Many existing systematic reviews and/or meta-analyses often focused on a single condition, either high BMI or ED, and very few provided further insights into the potential effectiveness of interventions that adopt an integrated approach to address both disorders concurrently [20]. The first systematic review and meta-analysis that investigated the potential effectiveness of preventive interventions of EDs on high BMI-related outcomes found that there is emerging evidence of some interventions that can be effective in preventing both disordered eating behaviours and unhealthy weight gain [21]. For example, the Healthy Weight program demonstrated reduction in both ED symptoms and BMI in females at up to three-year follow-up [21]. Several obesity prevention interventions have also reported positive effects on ED outcomes. For example, the Planet Health Program, a school-based intervention designed to promote good nutrition, physical activity and screen time behaviours, halved the incidence of unhealthy weight control behaviours (S. Bryn [22],S Bryn [23]. More recently, [20] conducted a systematic review to establish the evidence of “shared risk factors for obesity and eating disorders” demonstrated in prevention programs (i.e., programs that aimed to prevent both obesity and ED) in children and adolescents [20]. The narrative synthesis showed programs did not result in significant differences in weight status over time although these programs were found to reduce body dissatisfaction, dieting, and weight-control behaviors [20]. However, this systematic review did not conduct a meta-analysis to quantify the effectiveness of the integrated approach for prevention of both high BMI and EDs as well as limited scope to explore the potential effectiveness of preventive interventions for single condition, eight high BMI or ED, on another condition. There was a recent systematic review and meta-analysis that investigated the impact of obesity interventions on self-esteem and body image, however, it was limited to the paediatric population and only considered treatment-based interventions rather than preventive interventions [24]. As such, it is important to aggregate and synthesize the contemporary findings to inform the design of future interventions and public health policies for prevention of both obesity and EDs.

To our knowledge, there is no existing systematic review that summarises and quantifies the current evidence of the effectiveness of preventive interventions that potentially are effective in the prevention of both obesity and EDs across the age spectrum. Our review considered any preventive interventions that simultaneously reported ED and high BMI-related outcomes, regardless of the aim or design of the interventions (i.e. preventing high BMI, EDs or both). Importantly, this is the first systematic review that has incorporated a meta-analysis to quantify the effectiveness of interventions that are able to prevent both high BMI and EDs.

## Methods

This systematic review and meta-analysis adheres to the Preferred Reporting Items for Systematic Reviews and Meta-analyses (PRISMA) statement 2020 [25]. The protocol was published on PROSPERO (registration number CRD42020181575).

### Search strategy and selection criteria

A literature search was conducted using electronic databases that included MEDLINE, Embase, CINAHL complete, Global Health and APA PsycInfo from database inception to 10 May 2021. The search terms were organised into four concepts, including: (i) eating disorders; (ii) obesity/high BMI, OR physical activity OR fruit and vegetable intake; (iii) randomised controlled trial (RCT) or quasi-RCT; (iv) prevention or preventive interventions. Full search strategies are provided in the appendix (Table S1).

All citations were imported into an electronic database (Endnote® version X8), where duplicates were removed. A screening web-tool system, Covidence [26], was used for the screening process which was undertaken independently by two reviewers (OC and JP) using a four-stage blinded approach: (i) review of titles and abstracts; (ii) examination of full texts with respect to inclusion and exclusion criteria; and (iii) screening the reference lists of all included and in-scope studies to identify further eligible studies. Conflicts were resolved by a third reviewer (LL). Studies were included if they were randomised or quasi-randomised controlled trials that evaluated a preventive intervention (i.e. intervention targeted to those without an ED and/or overweight/obese) and reported both ED and BMI-related outcomes, regardless of study aim. The primary outcomes of interest for ED were body dissatisfaction, shape and weight concern, dieting, internalization and drive for thinness as these outcomes were found to be risk factors for high BMI-related outcomes as well as EDs. In this review, interventions that targeted individuals with clinically diagnosed EDs or overweight/obesity were not considered as preventive interventions. There were no restrictions regarding target population(s), year, or country of publication to ensure all potentially eligible studies were considered. Studies that were not peer-reviewed journal articles, published in languages other than English or did not provide a comparison between an intervention and control group were excluded (Table S2).

### Data extraction

All relevant characteristics of the studies were extracted into a standardised table that included: author’s name, year of publication, country, population characteristics, study arms, intervention duration and features, follow-up periods and results related to ED and high BMI outcomes. The studies were analysed in three groups according to the primary aim of the interventions: (i) ED prevention; (ii) high BMI prevention; and (iii) joint ED and high BMI prevention. The primary aim of a preventive intervention was determined from the intervention’s objectives, whether it focuses on ED prevention only or high BMI prevention only or both.

### Risk of bias assessment

The Cochrane risk-of-bias tool was used to evaluate the quality of included studies [27]. The assessment of the risk of bias required judgement of ‘high,’ ‘low’, or ‘unclear’ and was assessed by two independent reviewers (JP and ET). Domains were rated as ‘unclear’ if no information was provided, or insufficient detail was reported to allow an accurate assessment.

### Data analysis

A quantitative meta-analysis was performed to pool effects across the included studies using an Excel-based add-in package, MetaXL [28]. To determine the difference between the intervention group and the control group for ED and high BMI outcomes, an effect size was calculated, including confidence intervals (CIs). In addition to post-intervention, effect sizes were also calculated at follow-up periods (if available), which were categorized into two groups: (i) follow-up of up to 1 year and (ii) follow-up of more than 1 year. For studies that reported the same continuous outcome measured on the same scale, a weighted mean difference (WMD) was estimated. For studies that reported the same outcome measured using different scales, a Hedges’ g standardised mean difference (SMD) was estimated. Effect sizes of magnitude 0.2, 0.5 and 0.8 were considered small, moderate and large, respectively [29]. The quality-effects (QE) model was used to pool the data and was preferred over the conventional random effects (RE) model. Unlike the RE model, the QE model discounts studies with a higher risk of bias when redistributing inverse variance weights of individual studies in the meta-analysis [30, 31]. Only results from the QE model are presented except if there were differences in the direction of results between the two models; in this case, both QE and RE results are presented. The statistical heterogeneity of pooled studies was evaluated using the *I*^2^ and Cochran’s *Q* test statistics. Following the recommendations by the Cochrane Collaboration, any *I*^2^ statistic exceeding 40% and/or *Q* statistic being significant at *p* < 0.10 is indicative of substantial heterogeneity [27].

### Sensitivity analysis and publication bias

It was hypothesised ‘a priori’ that effectiveness may differ according to intervention types. Subgroup analyses were also performed by pooling the effects of studies with similar intervention type or technique including cognitive dissonance, CBT, healthy weight, physical activity, multicomponent, media literacy, psychoeducation, medication, CR and interpersonal therapy (IPT).

Two sensitivity analyses were undertaken to evaluate the robustness of the main meta-analysis results. First, an alternative model estimator, the inverse variance heterogeneity (IVhet), was used to pool the effects of different studies. A recent simulation study has shown that both the QE and IVhet models perform better than the RE model in minimising systematic/random errors and are able to generate estimates that are closer to the true population parameters [32]. Second, one-way sensitivity analyses were performed whereby individual studies were sequentially excluded from the meta-analysis. This method assesses whether effect sizes were driven by outliers and identified studies that were the prime determinants of the pooled results [28].

Further analysis was also carried out to determine the impact of potential unpublished or missed studies. Publication bias was assessed using funnel plots and Doi plots. Similar to funnel plots, Doi plots are interpreted based on their shape—symmetrical plots indicate a low risk of publication bias while asymmetrical plots indicate a high risk [28]. The Doi plots were also supplemented with the Luis Furuya–Kanamori (LFK) index of asymmetry, which was grouped into three categories of asymmetry (‘No asymmetry’, ‘Minor’, ‘Major’) [28].

## Results

**Fig. 1:**
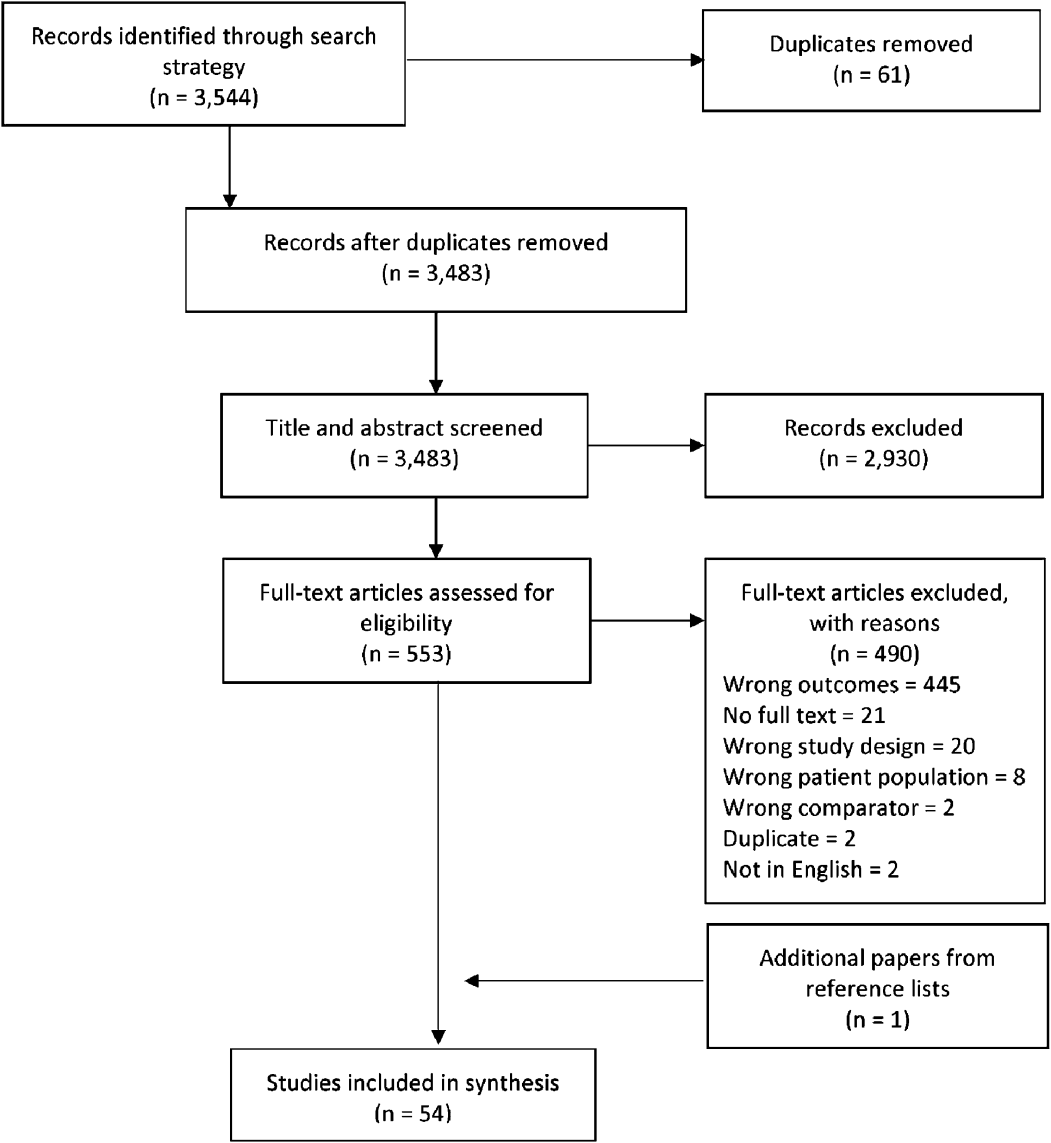
Preferred reporting items for systematic reviews and meta-analyses flowchart of systematic review results

A total of 3483 potentially relevant studies were identified after excluding duplicates (Fig. 1). After title and abstract screening, 553 studies were assessed as potentially meeting the inclusion criteria. Full text review resulted in 53 studies meeting the inclusion criteria, and the reference lists of the included studies yielded one additional paper. A total of 54 studies were included in our review.

### Characteristics of included studies

The number of studies with the primary aim of ED prevention (23/54) was similar to that of high BMI prevention (21/54), while there were only 10 studies that jointly aimed to prevent both ED and high BMI. The majority of studies were undertaken in the United States (57%), followed by Australia (9%) and the United Kingdom (7%). Studies were implemented across multiple settings, including schools (41%), universities (39%) and the broader community (20%). The majority of studies (34/54) focused on female participants. Most studies (approximately 85%) targeted children, adolescents and young adults (up to 24 years old).

Most preventive interventions involved a group-based format (36/54) and were delivered through face-to-face (F2F) (43/54), online (7/54) or a combination of F2F and online modalities (4/54). There were 11 trials that employed control groups with elements of minimal active intervention, the remaining studies used waitlist/delayed treatment or non-specified control that acted as a placebo. The intervention duration ranged from four weeks to three years, with the majority of interventions being delivered within a 12-month period (47/54). Interventions with more than a 12-month duration were mostly based in school settings where the intervention program was embedded within the curriculum and delivered over the subsequent academic year(s). The follow-up period ranged from one month to three years - most studies (37/54) did not conduct a follow-up or had a follow-up of up to one year. Intervention intensity varied according to the study’s primary aim. Studies with a primary aim of reducing high BMI, either alone or in combination with EDs, typically had a higher number of intervention sessions (e.g. > 10 sessions that can span multiple years) compared to studies that were primarily focused on preventing EDs which usually had fewer than 10 sessions. The ED outcomes investigated were varied, including dieting, body dissatisfaction, shape and weight concern, negative affect, ED symptoms, internalization and drive for thinness while the high BMI outcomes included BMI and physical activity. Further details and the characteristics of each study are presented in the appendix (Table S3).

### Qualitative synthesis of findings

#### Studies with a primary aim of ED prevention

The 23 studies with a primary aim of ED prevention included one or more of the following intervention techniques: cognitive dissonance (CD), cognitive behavioural therapy (CBT), multi-component, media literacy, psychoeducation, physical activity, medication, and others. Most of these studies (19/23) had evidence of positive impacts on ED outcomes (e.g. thin-ideal internalization, body dissatisfaction, dieting, shape and weight concern) at post-intervention to six-month follow-up, based on their key findings. A physical activity-based intervention (1/23) and a theory-based psychological intervention reported inconsistent findings. Three intervention techniques that included CD, CBT and multi-component (e.g. nutrition, and/or media literacy and/or physical activity) maintained a positive effect on ED outcomes (e.g. thin-ideal internalization, body dissatisfaction, dieting) at 6 to 12 months follow-up. For high BMI outcomes at six months post-intervention follow-up, most of the interventions (16/23) reported either inconsistent, insignificant or no impact on high BMI outcomes except for CD interventions (8/23) that reported a reduction in risk of obesity onset (estimated using Cox proportional hazards models). There was either no or inconsistent evidence to suggest that these interventions had positive effects on BMI beyond 6-months follow-up.

#### Studies with a primary aim of BMI prevention

Among the 21 studies with a primary aim of high BMI prevention, there were seven types of intervention techniques including CD, multi-component, healthy weight, psychoeducation, physical activity, behavioural therapy and others. The most promising intervention was the Healthy Weight program, which was evaluated in eight studies [33–39]. This intervention demonstrated reduction in high BMI outcomes (e.g. BMI, obesity and physical activity) and ED symptoms and risk factors (e.g. dieting, body dissatisfaction) up to 12-month follow-up in seven of the eight studies. School-based multi-component interventions were found to be effective in preventing both ED and high BMI post-intervention [22, 23, 40–44]. The remaining interventions reported inconsistent results (behavioural therapy) or did not find any change (psychoeducation and CD) on ED (e.g. ED symptoms, unhealthy weight loss practices, body image, emotional eating) and high BMI outcomes (e.g. BMI, physical activity, body fat). One study that involved counselling for pregnant women was found to have a positive impact on high BMI outcomes but increased body image concerns and depression [45].

#### Studies with a joint aim of ED and high BMI prevention

There were three types of interventions included in the 10 studies designated as joint preventive interventions for EDs and high BMI. Most of them were multi-component interventions (for example, physical activity, nutrition and motivational interview components were combined in a single study), followed by interpersonal psychotherapy and media literacy. Most of the interventions in this group (7/10) reported insignificant results in reducing ED outcomes (e.g. unhealthy weight control behaviours) except New Moves and Healthy Buddies. Regarding high BMI, no significant results were found in these studies. The only exception was a number of studies by Tanofsky-Kraff et al. who used interpersonal psychotherapy which resulted in a reduction in BMI at posttest, 6-month and 12-month follow-ups [46–48].

### Risk of bias assessment

Across the 54 studies included in this review, three studies had a low risk of bias across all domains, while 22 studies had a low risk of bias in at least five out of the eight domains. No studies were assessed to have a high risk of bias in all domains; however, five studies had a high risk of bias in at least three domains. Seven studies have an unclear risk of bias in at least half of the domains. All studies generally performed well in all domains except for participant and personnel blinding and allocation concealment where the proportion of studies with a low risk of bias was less than 50% (Figure S1). A summary of the risk of bias assessment is presented in the appendix (Table S4).

### Summary of meta-analysis results

There were 27 pooled comparisons between the intervention and control groups across 11 main outcomes, of which nine are related to eating disorders and two are related to high BMI. The pooled comparisons were conducted across all studies, regardless of their primary aim. The full details of these results are presented in Table 1.

**Table 1:** Summary of pooled effect results of the preventive interventions of eating disorders and high BMI

Outcome title	No. of studies at post-test (at follow-up)	No. of participants at post-test (at follow-up)	Statistical method	Pooled effect at post-test [95% CI] (*Q*, *p*, *I*^2^)	Pooled effect at up to 1-year follow-up [95% CI] (*Q*, *p*, *I*^2^)	Measure scale
Dieting						
All measures	15 (13) (5)	2631 (2218) (1219)	SMD (Hedges’g, QE, 95% CI) WMD (WMD, QE, 95% CI)	– **0.24 [** – **0.32, -0.16]** (*Q* = 24.05, *p* = 0.19, *I*^2^ = 21%)	– **0.24 [** – **0.31,** – **0.17]** (*Q* = 15.99, *p* = 0.52, *I*^2^ = 0%) * – 0.14 – 0.28, 0.00] (*Q* = 10.70, *p* = 0.10, *I*^2^ = 44%)	EDE-Q Restraint, DRES, DEBQ, DIET DRES
Shape and weight concern						
All measures	6 (5)	1346 (1259)	SMD (Hedges’g, QE, 95% CI)	– **0.42 [** – **0.69,** – **0.15]** *(Q* = *42.57, p* = *0.00, I*^*2*^ = *84%)*	– **0.45 [** – **0.63,** – **0.27]** *(Q* = *15.29, p* = *0.02, I*^*2*^ = *61%)*	McKnight RF, WCS, CIMEC
Weight concern scale (WCS)	3 (3)	590 (590)	WMD (WMD, QE, 95% CI)	– 9.37 [ – 19.19, 0.46] (*Q* = 17.08, *p* = 0.00, *I*^2^ = 88%)	– 5.92 [ – 9.33, – 2.52] (*Q* = 2.67, *p* = 0.26, *I*^2^ = 25%)	WCS
EDE-Q weight concern	2 (2)	199 (199)	WMD (WMD, QE, 95% CI)	– 0.20 [ – 0.49, 0.08] (*Q* = 0.00, *p* = 1.00, *I*^2^ = 0%)	– 0.30 [ – 0.58, – 0.02] (*Q* = 1.04, *p* = 0.31, *I*^2^ = 4%)	EDE-Q Weight Concern
EDE-Q shape concern	2 (2)	199 (199)	WMD (WMD, QE, 95% CI)	– 0.10 [ – 0.41, 0.21] (*Q* = 0.00, *p* = NAN, *I*^2^ = 0%)	– 0.29 [ – 0.58, 0.00] (*Q* = 0.88, *p* = 0.35, *I*^2^ = 0%)	EDE-Q Shape Concern
CIMEC	2 (2)	695 (669)	WMD (WMD, QE, 95% CI)	– 4.33 [ – 6.29, – 2.37] (*Q* = 8.44, *p* = 0.04, *I*^2^ = 64%)	– 5.69[ – 8.18, – 3.21] (*Q* = 10.84, *p* = 0.01, *I*^2^ = 72%)	CIMEC
Body dissatisfaction						
All measures	21 (16)	4827 (3597)	SMD (Hedges’g, QE, 95% CI)	– **0.21 [** – **0.30,** – **0.12]** *(Q* = *68.93, p* = *0.00, I*^*2*^ = *59%)*	– **0.17 [** – **0.24,** – **0.09]** (*Q* = 27.16, *p* = 0.17, *I*^2^ = 23%)	SDBPS, McKnight RF – body dissatisfaction, EDI-BD, EDI2-BD, EDI3-BD, MBAS, BSQ, NM, BSES-C, ABIS, DBI
Satisfaction and dissatisfaction with body parts scale (SDBPS)	11 (9) (6)	2021 (1523) (1700)	WMD (WMD, QE, 95% CI) WMD (WMD, QE, 95% CI)	– 0.32 – 0.43, – 0.21] (*Q* = 25.75, *p* = 0.03, *I*^2^ = 46%)	– 0.20 [ – 0.28 – 0.12] (*Q* = 10.76, *p* = 0.55, *I*^2^ = 0%) * – 0.12[ – 0.21, – 0.02] (*Q* = 16.82, *p* = 0.08, *I*^2^ = 41%)	SDBPS SDBPS
Eating disorder inventory-2 (EDI-2): body dissatisfaction	2 (3)	199 (856)	WMD (WMD, QE, 95% CI)	– 0.11 [ – 0.47, 0.26] (*Q* = 0.07, *p* = 0.79, *I*^2^ = 0%)	– 0.31 [ – 0.80, 0.18] (*Q* = 2.26, *p* = 0.32, *I*^2^ = 12%)	EDI-2 BD
Negative affect						
All measures	14 (12) (5)	2932 (2606) (1394)	SMD (Hedges’g, QE, 95% CI) SMD (Hedges’g, QE, 95% CI)	– **0.25 [** – **0.33,** – **0.19]** (*Q* = 18.51, *p* = 0.42, *I*^2^ = 3%)	– **0.13 [** – **0.22,** – **0.04]** (*Q* = 20.41, *p* = 0.16, *I*^2^ = 27%) * – 0.05 [ – 0.14, 0.04] (*Q* = 5.02, *p* = 0.83, *I*^2^ = 0%)	PANAS-X Sadness, PANAS-N, BDI, SADSSC, CESD PANAS-X Sadness, BDI, SADSSC
Eating disorder symptoms						
All measures	22 (19) (9)	4834 (4303) (2756)	SMD (Hedges’g, QE, 95% CI) SMD (Hedges’g, QE, 95% CI)	– **0.18 [** – **0.25,** – **0.12]** *(Q* = *40.29, p* = *0.08, I*^*2*^ = *28%)*	– **0.16 [** – **0.27,** – **0.06]** *(Q* = *37.63, p* = *0.07, I*^*2*^ = *31%)* * – **0.61 [** – **0.29,** – **0.04]** *(Q* = *42.14, p* = *0.00, I*^*2*^ = *67%)*	EDE-Q Bulimic Symptoms, EDI Bulimia, EDDI, EDDS, Global EDE-Q, ChEAT, EAT-26, EES, TFEQ, EDE-Q Eating Concern EDE-Q Bulimic Symptoms, EDDI, EAT-26
Eating disorder examination–questionnaire (EDE-Q): bulimic symptoms	3 (3)	641 (641)	WMD (WMD, QE, 95% CI)	– 0.12 – 0.24, 0.01] (*Q* = 12.25, *p* = 0.03, *I*^2^ = 59%)	– 0.10 [ – 0.18 – 0.02] (*Q* = 6.90, *p* = 0.23, *I*^2^ = 28%)	EDE-Q BS
Eating disorder inventory (EDI): Bulimia	2 (2)	493 (493)	WMD (WMD, QE, 95% CI)	– 0.03 [ – 0.74, 0.68] (*Q* = 6.59, *p* = 0.01, *I*^2^ = 85%)	– 0.15 [ – 0.36, 0.07] (*Q* = 0.56, *p* = 0.45, *I*^2^ = 0%)	EDI B
Eating disorder diagnostic interview (EDDI)	8 (7) (6)	1731 (1425) (1583)	WMD (WMD, QE, 95% CI) WMD (WMD, QE, 95% CI)	– 1.22 [ – 1.82, – 0.63] (*Q* = 8.10, *p* = 0.62, *I*^2^ = 0%)	-0.67 [-1.40, 0.06] (*Q* = 6.96, *p* = 0.64, *I*^2^ = 0%) * – 1.02 [ – 1.68, – 0.37] (*Q* = 3.09, *p* = 0.93, *I*^2^ = 0%)	EDDI EDDI
Eating disorder diagnostic scale (EDDS)	2 (1)	161 (95)	WMD (WMD, QE, 95% CI)	– 0.76 [ – 5.94, 4.42] (*Q* = 1.11, *p* = 0.29, *I*^2^ = 10%)	1.30 [ – 5.23, 7.85] (*Q* = 0.00, *p* = NAN, *I*^2^ = 0%)	EDDS
Eating attitudes test-26 (EAT-26)	3 (3)	886 (799)	WMD (WMD, QE, 95% CI)	– 2.24 [ – 4.86, 0.38] (*Q* = 22.12, *p* = 0.00, *I*^2^ = 82%)	– 2.74 [ – 5.71, 0.22] (*Q* = *22.20, p* = *0.00, I*^*2*^ = *82%)*	EAT-26
Eating disorder examination-questionnaire (EDE-Q): eating concern (EDE-Q EC)	2 (2)	199 (199)	WMD (WMD, QE, 95% CI)	0 [ – 0.13, 0.13] (*Q* = 0.00, *p* = n/a, *I*^2^ = 0%)	– 0.04 – 0.22, 0.13] (*Q* = 1.25, *p* = 0.26, *I*^2^ = 20%)	EDE-Q Eating Concern
Anthropometric						
All measures	18 (15) (8)	2835 (2852) (2274)	SMD (Hedges’g, QE, 95% CI) SMD (Hedges’g, QE, 95% CI)	0.02 [ – 0.05, 0.09] (*Q* = 20.18, *p* = 0.45, *I*^2^ = 1%)	– 0.03 [ – 0.11, 0.05] (*Q* = 23.34, *p* = 0.22, *I*^2^ = 19%) * – 0.04 [ – 0.14, 0.05] (*Q* = 20.42, *p* = 0.12, *I*^2^ = 31%)	BMI, Weight change, Percent body fat, Waist circumference, Age-adjusted percentile BMI, Weight change, Age-adjusted percentile
BMI	16 (15) (6)	2539 (2790) (1758)	WMD (WMD, QE, 95% CI) WMD (WMD, QE, 95% CI)	0.01 [ – 0.24, 0.27] (*Q* = 18.01, *p* = 0.46, *I*^2^ = 0%)	– 0.13 [ – 0.45, 0.18] (*Q* = 24.52, *p* = 0.10, *I*^2^ = 31%) *-0.33 [ – 0.77, 0.11] (*Q* = 15.54, *p* = 0.16, *I*^2^ = 29%)	BMI BMI
Percent body fat	2	409	WMD (WMD, QE, 95% CI)	– 0.21 [ – 1.86, 1.44] (*Q* = 0.53, *p* = 0.77, *I*^2^ = 0%)		Percent body fat
Waist circumference	2	106	WMD (WMD, QE, 95% CI)	1.21 [ – 7.91, 10.33] (*Q* = 3.86, *p* = 0.05, *I*^*2*^ = 74%)		Waist circumference
Physical activity						
All measures	5 (3)	916 (611)	SMD (Hedges’g, QE, 95% CI)	0.21 [ – 0.10, 0.53] (*Q* = 23.11, *p* = 0.00, *I*^*2*^ = 78%)	– 0.13 [ – 0.29, 0.03] (*Q* = 2.60, *p* = 0.63, *I*^2^ = 0%)	Accelerometer, IPAQ, HPLP, PYLPAS, PAQ
Unhealthy behaviour						
All measures	3	311	SMD (Hedges’g, QE, 95% CI)	– 0.15 [ – 0.53, 0.23] (*Q* = 5.21, *p* = 0.07, *I*^*2*^ = 62%)		BWCP, SCI-DSM IV, EDE
Internalization						
All measures	10 (8)	2279 (1781)	SMD (Hedges’g, QE, 95% CI)	– **0.42 [** – **0.58,** – **0.27]** (*Q* = 43.03, *p* = 0.00, *I*^*2*^ = *67%)*	– **0.25 [** – **0.34,** – **0.15]** (*Q* = 14.39, *p* = 0.28, *I*^2^ = 17%)	SATAQ-3 Internalization General, IBSS thin-ideal internalization
Sociocultural attitudes toward appearance questionnaire - 3 (SATAQ-3): internalization General (IG)	2 (2)	729 (603)	WMD (WMD, QE, 95% CI)	– 0.22 [ – 2.14, 1.71] (*Q* = 1.02, *p* = 0.31, *I*^2^ = 2%)	– 1.83 [ – 4.13, 0.48] (*Q* = 1.13, *p* = 0.29, *I*^2^ = 12%)	SATAQ-3 Internalization General
Ideal-body stereotype scale-revised (IBSSR) (thin-ideal internalization)	8 (6) (5)	1550 (1178) (1302)	WMD (WMD, QE, 95% CI) WMD (WMD, QE, 95% CI)	– 0.34 – 0.40, – 0.29] (*Q* = 9.50, *p* = 0.58, *I*^2^ = 0%)	– 0.18 [ – 0.26, – 0.10] (*Q* = 14.41, *p* = 0.16, *I*^2^ = 31%) *-0.12 [-0.18, -0.05] (*Q* = 12.95, *p* = 0.16, *I*^2^ = 31%)	IBSS thin-ideal internalization
Drive for thinness						
All measures	5 (4)	1446 (1053)	SMD (Hedges’g, QE, 95% CI)	– 0.27 – 0.59, 0.05] *(Q* = *27.11, p* = *0.00, I*^*2*^ = *85%)*	– **0.30 [** – **0.49,** – **0.11]** *(Q* = *5.61, p* = *0.13, I*^*2*^ = *47%)*	EDI3-DT, EDI-DT, EDI2-DT
EDI DT	2	710	WMD (WMD, QE, 95% CI)	– 1.93 [ – 5.94, 2.06] (*Q* = 24.10, *p* = 0.00, *I*^2^ = 96%)		EDI-DT
EDI-2 DT	2 (2)	199 (199)	WMD (WMD, QE, 95% CI)	– 0.26 [ – 2.93, 2.42] (*Q* = 3.66, *p* = 0.06, *I*^2^ = 73%)	– 0.47 [ – 3.90, 2.95] (*Q* = 5.26, *p* = 0.02, *I*^2^ = 81%)	EDI2-DT
Self-esteem						
All measures	2 (3)	729 (1260)	WMD (WMD, QE, 95% CI)	0.33 [ – 0.61, 1.27] (*Q* = 0.29, *p* = 0.59, *I*^2^ = 0%)	0.34 [ – 0.45, 1.13] (*Q* = 2.66, *p* = 0.26, *I*^2^ = 25%)	RSES

Significant results are in bold

*MD* standardized mean difference, *WMD* weighted mean difference, *QE* quality effect model, *EDE-Q* eating disorder examination—questionnaire, *dres* dutch restrained eating scale, *DEBQ* dutch eating behavior questionnaire, *DIET* diet and preoccupation with food. *McKnight RF* McKnight risk factor survey - overconcerns with weight and shape subscale, *WSC* weight and shape concern, *CIMEC* questionnaire on influences of aesthetic body ideal. *SDBPS* satisfaction and dissatisfaction with body parts scale, *EDI-BD* eating disorder inventory – body dissatisfaction scale, *MBAS* male body attitudes scale, *BSQ* body satisfaction questionnaire, *NM* new moves study, *BSES-C* body self-esteem scale for children, *ABIS* attitude towards body image scale, *DBI* distress about body image. *PANAS-X* the positive affect and negative affect scale-revised, *BDI* beck depression inventory, SADSSC schedule for affective disorders and schizophrenia for school-age children, *CESD* the center for epidemiologic studies—depression scale. *EDI* eating disorder inventory; *EDDI* the eating disorder diagnostic interview, *EDDS* the eating disorder diagnostic survey. *BMI* body mass index, *IPAQ* International Physical Activity Questionnaire, *HPLP* health promoting lifestyle profile, *PYLPAS* Past Year Leisure Physical Activity Scale, *PAQ* Paffenbarger Activity Questionnaire. *BWCP* behavioral weight control practices checklist, *SCI-DSM IV* structured clinical interview for DSM IV axis1 disorders, *ED* diagnostic section, *EDE* eating disorder examination—episodes in the past 28 days. *SATAQ* sociocultural attitudes towards appearance questionnaire, *IBSS* the Ideal-Body Stereotype Scale, *EDI-DT* eating disorder inventory—drive for thinness scale. *RSES* Rosenberg Self-esteem Scale. *ChEAT* eating attitudes test—children version, *EAT-26* eating attitudes test-26, *EES* Emotional Eating Scale, *TFEQ* the Three-Factor Eating Questionnaire

Figures in parentheses are at follow-up

Q = Cochran’s Q test statistic

p = p value for Q

I^2^ = I squared test statistic

^*^At second follow-up: > 1 year of follow-up

The pooled comparison for restrictive dieting measures from 15 studies indicated a small but significant effect size at post-intervention (g – 0.24, 95% CI: – 0.32 to – 0.16) and the immediate follow-up. Similarly, the pooled comparison for negative affect measures suggested a small but significant effect size at post-intervention (g – 0.25, 95% CI:  – 0.32 to – 0.18) and first follow-up (g – 0.11, 95% CI – 0.18 to – 0.05).

The pooled comparison for shape and weight concern measures from six studies suggested a significant and moderate effect size at post-intervention (g – 0.42, 95% CI: – 0.69 to – 0.15) and follow-up but there was considerable heterogeneity across studies. Analysis for measures related to body dissatisfaction, ED symptoms and internalization also revealed a similar pattern - significant differences between intervention and control groups at post-intervention and follow-up but substantial heterogeneity across studies.

There appeared to be no significant differences between the intervention and control groups for the remaining ED-related outcomes, namely unhealthy behaviour (three studies), drive for thinness (five studies) and self-esteem (two studies), with the exception of drive for thinness measures at follow-up (four studies) which showed a small effect size (g – 0.30, 95% CI: – 0.49 to – 0.11).

For anthropometric measures (18 studies), there were no significant differences between the intervention and control groups at post-intervention (g 0.02, 95% CI:  – 0.05 to 0.09) or subsequent follow-ups. The trend is similar when individual measures such as body mass index, percent body fat and waist circumference were examined in isolation. The pooled comparison for another high BMI-related outcome, physical activity, also suggest no significant differences between the intervention and control groups.

### Subgroup analyses and sensitivity analyses

The subgroup analysis, as presented in Table 2, indicated that the small-to-moderate effect on dieting and negative affect measures remained significant at 1-year follow-up for two main intervention techniques—CBT and CD. When comparing the differences between the intervention and control groups at post-intervention, CD-based and Healthy Weight interventions had small-to-moderate impacts on improvements in body dissatisfaction and ED symptoms. The subgroup analysis did not reveal any significant effect size for high BMI-related outcomes in any type of intervention, including CBT, CD and Healthy Weight. The pooled comparisons for other intervention techniques including media literacy, psychoeducation, medication, IPT and others was not possible due to the relatively small number of studies available to pool.

**Table 2:** Pooled comparisons between intervention and control groups by intervention technique

Outcome	Time	Cognitive dissonance	Cognitive behavioural therapy	Healthy weight	Physical activity	Multi-component
Dieting	Post-test	– **0.29 (** – **0.39,** – **0.19)**	– **0.46 (** – **0.82,** – **0.09)**	– 0.11 ( – 0.26, 0.04)	–	–
	1-Year FU	– **0.26 (** – **0.38,** – **0.14)**	– **0.43 (** – **0.67,** – **0.19)**	– **0.19 (** – **0.33, ** – **0.06)**	–	–
Shape and weight concern	Post-test	–	– 0.62 ( – 1.26, 0.04)	–	–	–
	1-Year FU	–	– **0.36 (** – **0.53,** – **0.18)**	–	–	–
Body dissatisfaction	Post-test	– **0.41 (** – **0.59,** – **0.22)**	– 0.11 ( – 0.35, 0.13)	– **0.28 (** – **0.41,** – **0.15)**	0.06 ( – 0.06, 0.18)	– **0.16 (** – **0.28,** – **0.04)**
	1-Year FU	– **0.28 (** – **0.39,** – **0.16)**	– 0.21 ( – 0.45, 0.03)	– 0.12 ( – 0.25, 0.01)	–	– 0.05 ( – 0.20, 0.10)
Negative affect	Post-test	– **0.35 (** – **0.47,** – **0.23)**	– 0.15 ( – 0.32, 0.02)	– **0.27 (** – **0.43, ** – **0.10)**	–	–
	1-Year FU	– **0.18 (** – **0.32,** – **0.04)**	– **0.18 (** – **0.34,** – **0.01)**	0.03 ( – 0.12, 0.17)	–	–
ED symptoms	Post-test	– **0.20 (** – **0.31,** – **0.10)**	– 0.14 ( – 0.49, 0.20)	– **0.22 (** – **0.32, ** – **0.12)**	–	– 0.15 ( – 0.40, 0.09)
	1-Year FU	– 0.09 ( – 0.22, 0.04)	– 0.13 ( – 0.28, – 0.02)	– **0.14 (** – **0.24, ** – **0.03)**	–	– 0.24 ( – 0.59, 0.11)
Unhealthy behaviour	Post-test	–	–	–	–	–
	1-Year FU	–	–	–	–	–
Internalization	Post-test	– **0.60 (** – **0.70,** – **0.49)**	–	–	–	–
	1-Year FU	– **0.26 (** – **0.40,** – **0.11)**	–	–	–	–
Drive for thinness	Post-test	–	– **0.54 (** – **0.83,** – **0.24)**	–	–	–
	1-Year FU	–	– **0.41 (** – **0.57,** – **0.24)**	–	–	–
Self-esteem	Post-test	–	–	–	–	–
	1-Year FU	–	–	–	–	0.34 ( – 0.45, 1.13)
Anthropometric (including BMI)	Post-test	0.09 ( – 0.06, 0.24)	– 0.01 ( – 0.18, 0.16)	0.02 ( – 0.15, 0.19)	–	–
	1-Year FU	0.00 ( – 0.21, 0.22)	0.00 ( – 0.17, 0.17)	0.01 ( – 0.16, 0.18)	–	–
Physical activity	Post-test	–	–	–	–	–
	1-Year FU	–	–	–	–	–

The pooled comparisons between intervention and control groups for intervention techniques of media literacy, psychoeducation, medication, cognitive reappraisals, interpersonal psychotherapy and others were not possible because the number of studies available to pool was less than 3. Numbers in bracket denote 95% CI. ‘Post-test’ refers to post-intervention period and ‘1-year FU’ refers to a follow-up of up to 1 year. Cells with ‘–‘ denote effect size was not calculated because the number of studies available to pool was less than 3

Significant results are in bold

The first sensitivity analysis, which involved using a different model estimator (IVhet) to calculate effect sizes, produced outcomes that were consistent with the main results. Most of the effect size estimated using IVhet (including CIs) for all 11 main outcomes were very similar to those using QE—the largest difference between the two model estimators for an effect size was less than 0.03 standard deviations. The second sensitivity analysis was the sequential exclusion of studies when calculating the effect size between the intervention and control groups for each of the 11 main outcomes. The results remain qualitatively similar for eight main outcomes, indicating that the impact of outliers is likely to be minimal. These outcomes were dieting, body dissatisfaction, negative affect, ED symptoms, anthropometric, physical activity, internalisation and self-esteem. On the other hand, the results for the remaining three main outcomes were affected when certain studies were not included in the calculation. The effect size for shape and weight concern at post-intervention became insignificant when Gonzalez et al. (2011) was excluded (g – 0.26, 95% CI:  – 0.56 to 0.04). The effect sizes for unhealthy behaviour and drive for thinness at post-intervention turned significant when certain studies were excluded; for the former, West et al. [39] (g – 0.28, 95% CI:  – 0.53 to – 0.04) and for the latter, Zabinski et al. (2001) (g – 0.35, 95% CI: – 0.69 to – 0.01).

### Publication bias

Based on visual inspection of Doi plots and the LFK index, there was a low risk of publication bias observed for most of the pooled effect results. The shape and weight concern and unhealthy behaviour outcomes reported by the included studies, however, exhibited a clear asymmetrical plot and a high LFK index, indicating a high risk of publication bias. Likewise, the results for dieting, body dissatisfaction and internalisation suggest potential bias, but these were only found at later follow-up periods. S5 Table in the appendix provides further details of the risk of publication bias for all outcomes.

## Discussion

Our review identified a large number of relatively good quality preventive intervention studies that report both high BMI and ED outcomes. Meta-analysis results indicate that the included preventive interventions had a moderate-to-large effect on several ED outcomes. These included dieting, shape and weight concerns, body dissatisfaction, negative affect, eating disorder symptoms and internalisation at post-intervention; with effects maintained up to one-year follow-up. Regarding high BMI outcomes, no effects were found for anthropometric or physical activity measures. Our review shows that there is a low risk of publication bias for most of the pooled effect results. It worth noting that there are some promising interventions that prevent both obesity and ED outcomes. For example, the Planet Health program which is delivered within the school setting was shown to half the prevalence of obesity and unhealthy weight control behaviours among females [49] while New Moves was able to reduce unhealthy weight control behaviours and sedentary behaviours and increase physical activity [50]. Project Health uses cognitive dissonance techniques to encourage healthy lifestyle behaviors and was effective in reducing obesity onset and ED onset over a 2-year follow-up period [34]. Interpersonal psychotherapy was demonstrated to reduce loss of control eating and BMI in teenager girls.

To our knowledge, this is the first comprehensive review to establish the evidence of effectiveness of preventive interventions for high BMI and EDs across the age spectrum. Most interventions included in this review were found to be effective in improving ED outcomes, but not high BMI outcomes. Changes in BMI are generally harder to achieve over shorter durations than changes in ED symptoms. Therefore, in addition to BMI, changes in diet and physical activity behaviours should be considered as intermediate outcomes of interest in future studies [51]. Our qualitative results are consistent with results from a recent narrative review of preventive interventions for obesity and EDs among adolescents that reported no significant effect on weight status [20]. A previous review we conducted of preventive interventions for eating disorders has shown that the Healthy Weight program was able to reduce both ED risk factors/symptoms and BMI [21]. However, with the inclusion of an additional and recent evaluation of the Healthy Weight program in this review [34], the previously observed significant effect on BMI was not replicated and failed to reach significance in our meta-analysis. While the reason for this reversal of results is unclear, it is worth noting that a new version of the Healthy Weight program, Project Health, which was captured in our review was more effective compared to the Healthy Weight program and control group in reducing ED symptoms, BMI and obesity onset in a recent RCT. Furthermore, there is some school-based obesity prevention interventions such as Planet Health or New Moves demonstrating the effectiveness in reducing BMI or obesity onset and unhealthy weight control behaviours. These interventions may be promising in preventing both eating disorders and high BMI, however, these interventions were not examined in the meta-analyses as the relevant data were unavailable. Given the inconclusive evidence on high BMI outcomes, further research is necessary to explore the risk factors that are shared by ED and high BMI and the most effective method of addressing them jointly. In this context, interventions targeting shared upstream determinants including exposure to food environments, food insecurity [52] and physical activity environments that encourage disordered eating and low levels of physical activity may be helpful and should be considered in future studies [53].

An important limitation in our effort to summarise the overall evidence of preventive interventions for high BMI and EDs is the heterogeneity of outcome measures across the included studies. For example, in the subgroup analysis, we were unable to pool results from interventions with the primary aim of prevention of high BMI only or combination given that the ED outcomes were measured inconsistently (dieting vs body dissatisfaction vs loss of eating control). Although studies targeting ED prevention use the BMI as the main outcome index, the measurement of BMI alone in studies included in our review may be inadequate to capture full benefit of the intervention on prevention of high BMI as studies require long-term follow-up in order to demonstrate impact on BMI. It is noted that no effect on BMI outcomes in studies evaluated preventive interventions for ED outcomes might be a positive sign to indicate no adverse effect on obesity outcomes. However, inconsistent validated tools to measure EDs in studies with the primary aim of prevention of high BMI leads to uncertainty in relation to whether preventive interventions for high BMI have any benefit or side effect on EDs. The possible harmful consequences of obesity prevention initiatives on the development of disordered eating (such as body image, dieting, weight-related teasing, excessive weight preoccupation) have been raised in the literature [18, 54]. For example, periodic assessments of BMI—with the aim of identifying overweight children and reporting back to parents by letter, advising dietary changes and physical activity that are already in place in some states in the USA and Australia, if not applied carefully, could potentially have unintentional effects, such as parents encouraging their child to diet and increased stigmatization of obesity [54]. Interestingly, in this review, we found that counselling for pregnant women was found to have a positive impact on high BMI outcomes but increased body image concerns and depression. Further research should establish evidence to ensure attempts to curb high BMI or ED do more good than harm. It is important that researchers in the obesity and ED prevention should assess variables of interest for both fields.

### Strengths and limits

One of the strengths of our systematic review and meta-analysis is being the first comprehensive review of the effectiveness of preventive interventions on both high BMI and EDs across the age spectrum. Studies with the primary aim of either the prevention of high BMI or an ED were included as long as outcomes related to both these issues were reported. In addition, we also performed qualitative synthesis and quantitative subgroup analyses to investigate the effectiveness of preventive interventions by primary aim and technique of the intervention. Nevertheless, there are several other limitations with our review that need consideration. First, our review focused on evidence from RCTs or quasi-RCTs only. Pre–post or controlled trials without randomisation were excluded. Second, the trials included in this review and meta-analysis had to report both high BMI and ED-related outcomes. Therefore, any trials that evaluated the same intervention but reported only high BMI or ED outcomes were excluded. This might underestimate or overestimate the results of our meta-analysis. Third, our review may have missed studies which have been published in languages other than English, especially studies conducted in low-to-middle-income countries.

## Conclusion

The public health burden of high BMI and EDs has been well documented. This study observed that there is inconclusive evidence to indicate that preventive interventions have a positive impact on both conditions. Although some programs showed promising results in reducing both ED risk factors and obesity onset risks, further research is necessary to identify remedial risk factors that are shared by these weight-related disorders. Improved methodologies for outcome measurement and intervention designs are also needed to investigate potentially effective preventive interventions.

### What is already known on this subject?

Obesity is a common co-morbidity of eating disorders, particularly for bulimia nervosa and binge eating disorder. There are effective interventions that prevent either eating disorders or high body mass index. However, limited evidence is available on the effectiveness of interventions that prevent both conditions simultaneously.

### What does this study add?

There is preliminary evidence that several interventions have an impact on both eating disorders and high body mass index. However, this evidence is characterised by uncertainty due to a low number of randomised controlled trials in this area. Our review highlights the need for future research to guide the development of effective interventions that target both eating disorders and elevated body mass index.

## Supplementary Information

Below is the link to the electronic supplementary material.

Supplementary file1 (DOCX 271 KB)

## Data Availability

All relevant data are within the manuscript and its supporting information files.
