# Supplementary material for: Prevention of high body mass index and eating disorders: a systematic review and meta-analysis
Source: Eat Weight Disord. 2022 Aug 27;27(8):2989–3003. doi: 10.1007/s40519-022-01458-8 (PMC9803760; doi:10.1007/s40519-022-01458-8)
Supplement: Supplementary file 1 — Supplementary file1 (DOCX 271 KB) [file 40519_2022_1458_MOESM1_ESM.docx]

**Prevention of high body mass index and eating disorders: A systematic review**

Long Le ^1^, Eng Joo Tan ^1^, Jo Perez^1^, Oxana Chiotelis^1^, Phillipa Hay ^2,3^, Jaithri Ananthapavan^1,4^, Yong Yi Lee^1,5^, Cathrine Mihalopoulos^1^

^1^Deakin Health Economics, Institute for Health Transformation, School of Health and Social Development, Deakin University, Burwood, Victoria 3125, Australia (L Le PhD, EJ Tan PhD, J Perez, O Chiotelis, J Ananthapavan, YY Lee PhD, Prof C Mihalopoulos PhD)

^2^Health Research Institute (THRI), School of Medicine, Western Sydney University, Locked Bag 1797, Penrith, NSW 2751, Australia (Prof P Hay PhD)

^3^Camden and Campbelltown Hospital, SWSLHD, Campbelltown, NSW 2560, Australia (Prof P Hay PhD)

^4^Global Obesity Centre, Institute for Health Transformation, School of Health and Social Development, Deakin University, Burwood, Victoria 3125, Australia (J Ananthapavan)

^5^School of Public Health, The University of Queensland, Herston, QLD 4006, Australia (YY Lee PhD)

Correspondence to: Dr Long Le

Deakin Health Economics, Institute for Health Transformation,

School of Health and Social Development, Deakin University, Burwood, Victoria 3125, Australia

[long.le@deakin.edu.au](mailto:long.le@deakin.edu.au)

+61-392468383

**Table S1a. Search concepts and the corresponding key words used**

| Concept 1 |  | Eating disorders | eating disorder* OR (“weight control behavio*”) OR anorexi* OR bulimi* OR dieting OR ((shape* OR weight OR eating) N2 concern*)) OR (“thin-ideal internali?ation”) OR (“negative affect*”) OR (“body dissatisfaction”) |
| --- | --- | --- | --- |
| Concept 2 |  | Obesity/ high BMI  Physical activity Fruit and vegie intake | AND obes* OR overweight OR “weight related problem*” OR ((weight N2 (gain* OR change*)) OR overeat* OR “over eat*” OR BMI OR “body mass index” OR ((“body mass” N2 (gain* OR change*)) OR ((fruit* OR vegetable*) N2 (consump* OR intake)) OR ((kj OR kilojoules OR food) N2 (consum* OR intake)) OR (“physical* activ*”) OR (“physical* inactiv*”) OR (“metabolic equivalent*”) OR (“sedentary life*”) OR (“sedentary behavio*”) |
| Concept 3 |  | Type of studies | ((random* OR clinical OR control*) N2 trial) |
| Concept 4 |  | Preventions | ((health OR school* OR food OR nutrition) N2 (promot* OR educat* OR policy OR policies)) ) OR prevent* |
| Mesh term searching |  |  | (MH "Binge-Eating Disorder") OR (MH "Feeding and Eating Disorders+") OR (MH "Body-Weight Trajectory") OR (MH "Weight Loss") OR (MH "Anorexia") OR (MH "Anorexia Nervosa") OR (MH "Bulimia") OR (MH "Bulimia Nervosa") OR (MH "Diet Therapy") OR (MH "Weight Gain") OR (MH "Body Weight") OR (MH "Weight Reduction Programs") OR (MH "Body Image") OR (MH "Randomized Controlled Trial+") OR (MH "Clinical Trial Protocol") OR (MH "Controlled Clinical Trial+") OR (MH "Pragmatic Clinical Trial") OR (MH "Obesity") OR (MH "Overweight") OR (MH "Weight Gain") OR (MH "Body Weight Changes") OR (MH "Body Mass Index") OR (MH "Appetite Regulation") OR (MH "vegetable intake") OR (MH "Metabolic Equivalent") OR (MH "Sedentary Behavior") OR (MH "Healthy Lifestyle") OR (MH "Risk Reduction Behavior") OR (MH "Health Promotion") OR (MH "Health Policy") OR (MH "School Health Services") OR (MH "Health Education") OR (MH "Nutrition Therapy") OR (MH "Nutrition Policy"). |

**Table S1b**. Inclusion & exclusion criteria

| **Inclusion criteria** | **Exclusion criteria** |
| --- | --- |
| Primary aim of study was prevention of high body mass index, eating disorders or both;  Outcomes related to eating disorders and high BMI were reported;  Human studies only;  Access to full text;  Randomised or quasi-randomised controlled trials. | No control group to compare with the intervention group;  Interventions targeted at participants diagnosed with an eating disorder; or those with BMI over 25 kg/m^2^.  Focus on treatment options rather than prevention;  Not published in English |

**Figure S1.** Summary of risk of bias for included studies.


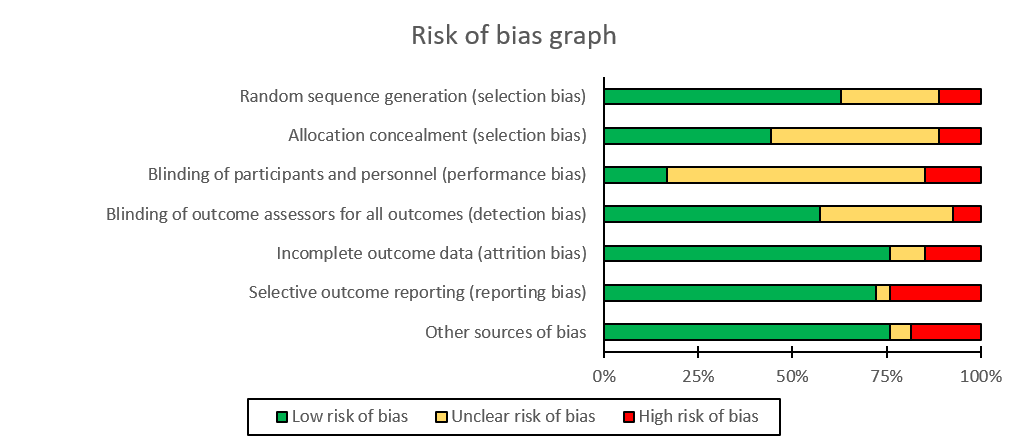


**Table S2a: Characteristics of included studies**

| **No** | **Study Country** | **Sample Mean Age or Range (SD)** | **Study Arms** | **Intervention Duration** | **Intervention Format** | **Follow up** | **Outcomes and Results** | |
| --- | --- | --- | --- | --- | --- | --- | --- | --- |
|  |  |  |  |  |  |  | **ED-related** | **High BMI-related** |
| **Primary Aim: Eating Disorder (ED) prevention** | | | | | | | | |
| 1 | Armitage et al. (2015)  UK | All women 18 and above  (M=28.48years, SD=11.31)  Setting: Community | 1. Theory-based psychological intervention  2. Control group testing | Not specified | individual, online questionnaire with volitional help sheet at the end, linking critical situations with appropriate response.    Intervention: drop down menus to make link and asked to form implementation intentions.  Control: tick box to make link only. | 1-month post baseline | Primary outcomes | Primary outcomes |
|  |  |  |  |  |  |  | Emotional Eating in response to:  ꓿Depression  ꓿Anxiety  ↓Boredom |  |
|  |  |  |  |  |  |  | Secondary outcomes | Secondary outcomes |
|  |  |  |  |  |  |  |  |  |
| 2 | Bearman et al. (2003)  USA | 74 females aged 17-20 (M=18.9); with body image concerns  Setting: Colleges/universities | 1. Cognitive behavioural intervention (CBT)  2. Wait-list control | 4 weeks | Group  four 1-hr sessions of 5-12 women | 4(posttest), 8, 16, 28 weeks    *week28 FU for CBT group only | Primary outcomes | Primary outcomes |
|  |  |  |  |  |  |  | ↓Body Dissatisfaction  =Negative Affect  ↓Depressive symptoms at 4 and 16weeks, but not at 8 and 28weeks  ↓Dieting  ↓Bulimic symptoms at 8, 16 but not at 28weeks | BMI - reported baseline data only |
|  |  |  |  |  |  |  | Secondary outcomes | Secondary outcomes |
|  |  |  |  |  |  |  |  |  |
| 3 | Jacobi et al. (2007)  Germany | Female students 18-29 years; BMI between 18-30; no ED diagnosis/treatment in the past year; no other psychopathology problems  Setting: Colleges/universities | 1. Internet-based intervention (adapted version of Student Bodies)  2. Waiting-list control | 8 weeks | Individual  8-weekly session online, with twice per week reminder if failed to log in. | Posttest and 3 months | Primary outcomes | Primary outcomes |
|  |  |  |  |  |  |  | Eating disorder examination-questionnaire (EDE-Q subscales)  ↓EDE-Q restraint*  ꓿EDE-Q eating concern  ↓EDE-Q weight concern  ↓EDE-Q shape concern    Eating disorder inventory 2 (EDI-2 subscale)  ↓EDI-2 drive for thinness  ꓿EDI-2 body dissatisfaction    ↓Weight concerns scale (WCS)*    *at post intervention only |  |
|  |  |  |  |  |  |  | Secondary outcomes | Secondary outcomes |
|  |  |  |  |  |  |  | ꓿SCL-90-R global severity index  DSM-IV -measured to exclude diagnosed with ED. Result not reported | ꓿BMI |
| 4 | Jacobi et al. (2012)  Germany | Women aged 18-35 years (M=22.3; SD=2.9); 17.5 < BMI < 33; scoring >42 on the Weight Concern Scale; with subthreshold ED symptoms  Setting: Colleges/universities | 1. Student Bodies+ (SB+) intervention  2. Wait-list control group | 8 weeks | 8- weekly session online, with moderated  discussion group | Posttest and 6 month | Primary outcomes | Primary outcomes |
|  |  |  |  |  |  |  | ↓Binges  ↓Purging  ꓿Restrictive Eating    EDE-Q subscales  ↓Restraint  ↓Eating concern  ↓Weight Concern  ↓Shape concern  ↓Total | ↓BMI |
|  |  |  |  |  |  |  | Secondary outcomes | Secondary outcomes |
|  |  |  |  |  |  |  | ↓Weight Concern Scale (WCS)  EDI-2 subscales  ↓Drive for thinness  ↓Bulimia  ↓Body dissatisfaction   ↓Depression (BDI)*  ↓Brief Symptom Inventory (GSI)*    * only at post intervention |  |
| 5 | Kass et al. (2014)  USA | College women aged 18-25 years; WCS score at or above 47; did not meet criteria for clinical/subclinical ED; not suicidal or psychotic  Setting: Colleges/universities | 1. Student Bodies with a guided discussion group  2. Student Bodies without guided discussion group | 8 weeks | 8week online session with 1 session released at the start of the week. Average of 21 pages length of lesson each session; with guided group discussion for the first intervention arm(SB+guided) | Posttest | Primary outcomes | Primary outcomes |
|  |  |  |  |  |  |  | ꓿EDE-Q Binge Episodes  ↓Weight concern scale (WCS)  Depression (BDI-II)*    *only reported baseline data | BMI* |
|  |  |  |  |  |  |  | Secondary outcomes | Secondary outcomes |
|  |  |  |  |  |  |  |  |  |
| 6 | Lopez-Guimera et al. (2011)  Spain | Grade 8 female students (M=13.41, SD=0.38)  Setting: Secondary schools | 1. Nutrition and Media literacy intervention (NUT + ML)  2. Media literacy intervention (ML)  3. Non-treatment control group (NT) | 5 weeks | Group  NUT: one 90-minute sessions  ML: two 90-minute sessions plus two 60-minute activity sessions, one session per week for 4 weeks.  NUT+ML: one session per week for 5 weeks  F2F/Class | Posttest and 6 months | Primary outcomes | Primary outcomes |
|  |  |  |  |  |  |  | NUT+ML vs NT  ꓿Eating Attitudes Test (EAT)   ꓿Diet and preoccupation with food (DIET)  ↓Influences on body shape model-26 (CIMEC-26)   ꓿Distress about Body Image (DBI)    NUT+ML vs ML  ꓿Eating Attitudes Test (EAT)   ꓿Diet and preoccupation with food (DIET)  ꓿Influences on body shape model-26 (CIMEC-26)   ꓿Distress about Body Image (DBI)    ML vs NT  ꓿Eating Attitudes Test (EAT)  ↓Diet and preoccupation with food (DIET)  =Influences on body shape model-26 (CIMEC-26)   ꓿Distress about Body Image (DBI)꓿Distress about Body Image (DBI) | BMI not reported |
|  |  |  |  |  |  |  | Secondary outcomes | Secondary outcomes |
|  |  |  |  |  |  |  |  |  |
| 7 | O'Brien & LeBow (2007)  Canada | Female university students  (M=22.2 years,   SD not reported)  Setting: Colleges/universities | 1. Experimental condition (EX) (psychoeducational intervention)  2. Self-monitoring control condition (SMC) | 8 weeks | Group  8 sessions 60-90min each  F2F | Posttest, 3 and 6 months | Primary outcomes | Primary outcomes |
|  |  |  |  |  |  |  | ↓Daily meal record  =Daily exercise record  ↓Forbidden Food Survey  ↓Bulimia Test-Revised  ↓Body Shape Questionnaire  ↓Eating Attitudes Test-26 | =BMI |
|  |  |  |  |  |  |  | Secondary outcomes | Secondary outcomes |
|  |  |  |  |  |  |  | =Beck Depression Inventory-II |  |
| 8 | Olive et al. (2019)  Australia | School children  (M=8 years,   SD=0.3)  Setting: Primary schools | 1. Specialist-taught PE  2. Control | 4 school years | Group  2 x 50min lessons per week  F2F | 1 and 4 years | Primary outcomes | Primary outcomes |
|  |  |  |  |  |  |  | ↓Depression*  =Stress  ↓ Body dissatisfaction*    *effect not sustained at 4 years | BMI, physical activity and percent body fat reported at baseline only and used as covariates. |
|  |  |  |  |  |  |  | Secondary outcomes | Secondary outcomes |
|  |  |  |  |  |  |  |  |  |
| 9 | Plessow et al. (2018)  USA | Oligo-amenorrheic female athletes  (M=19.8 years,  SD=0.2)  Setting: Community | 1. PATCH (estradiol patch)  2. PILL (combined oral contraceptive pill)  3. E+ (with estrogen, PATCH + PILL)  4. E- (no estrogen) | 12 months | Individual  medication  100 bearmcg transdermal 17β-estradiol with cyclic progesterone (PATCH), 100 mcg transdermal 17β-estradiol with cyclic progesterone (PILL) for 12 months | Posttest | Primary outcomes | Primary outcomes |
|  |  |  |  |  |  |  | PATCH vs E-  ↓Drive for thinness  ↓Body dissatisfaction  =Cognitive restraint  =Uncontrolled eating  =Emotional eating    PILL vs E-  =Drive for thinness  =Body dissatisfaction  =Cognitive restraint  =Uncontrolled eating  =Emotional eating    E+ vs E-  ↓ Drive for thinness  ↓ Body dissatisfaction  =Cognitive restraint  =Uncontrolled eating  =Emotional eating | PATCH vs E-  =Weight  =BMI  =BMI z-score  =Total daily caloric intake      PILL vs E-  =Weight  =BMI  =BMI z-score  =Total daily caloric intake    E+ vs E-  =Weight  =BMI  =BMI z-score  =Total daily caloric intake |
|  |  |  |  |  |  |  | Secondary outcomes | Secondary outcomes |
|  |  |  |  |  |  |  |  |  |
| 10 | Sanchez-Carracedo et al. (2016)  Spain | School children  (M=13.83 years,  SD=0.53)  Setting: Secondary schools | 1. Universal disordered eating intervention (based on social cognitive model, media literacy educational approach and cognitive dissonance theory)  2. Control | 8 weeks | Group  60-min weekly session  F2F | Posttest, 1 year | Primary outcomes | Primary outcomes |
|  |  |  |  |  |  |  | ↓SATAQ-3 Internalization general  ↓SATAQ-3 Pressures  ↓Children eating attitudes test   ↓Perception of teasing scale  =Body dissatisfaction  =Drive for thinness   =Negative affect scale  =Rosenberg self-esteem scale | =BMI |
|  |  |  |  |  |  |  | Secondary outcomes | Secondary outcomes |
|  |  |  |  |  |  |  |  |  |
| 11 | Stice et al. (2001)  USA  (A randomized trial of a dissonance-based eating disorder prevention program)*    *only results for the ED-focused interventions in this study are presented here | Female young adults  (Age range 17-29 years, mean age and SD not reported)  Setting: Colleges/universities | 1. Dissonance-based intervention  2. Healthy weight management control | 3 weeks | Group  3 weekly 1-hour sessions  F2F | Posttest, 4 weeks | Primary outcomes | Primary outcomes |
|  |  |  |  |  |  |  | ↓Thin-Ideal internalization  ↓Body dissatisfaction  =Dieting  =Negative affect  =Bulimic symptoms  =Fat consumption | =BMI  =Exercise behaviours |
|  |  |  |  |  |  |  | Secondary outcomes | Secondary outcomes |
|  |  |  |  |  |  |  |  |  |
| 12 | Stice et al.  (2002)   USA | Female undergraduates  (Age range 17-55, mean age and SD not reported)  Setting: Colleges/universities | 1. Dissonance-based intervention  2. Control | 4 months | Group  1.5hr class seminar twice weekly over the semester  F2F | Posttest | Primary outcomes | Primary outcomes |
|  |  |  |  |  |  |  | ↓Thin-ideal internalization (IBSS-R)  ↓Body dissatisfaction  ↓Dieting (DRES)  ꓿Depressive symptoms (BDI-II)  ↓Eating disorder symptoms | ↓BMI |
|  |  |  |  |  |  |  | Secondary outcomes | Secondary outcomes |
|  |  |  |  |  |  |  |  |  |
| 13 & 14 | Stice et al. (2006a)  USA  (Journal of Consulting and Clinical Psychology)*  Stice et al. (2008a)  USA  (Journal of Consulting and Clinical Psychology)*    *only results for the ED-focused interventions in this study are presented here | Adolescent girls  (M=17.0 years,  SD=1.4)  Setting: Secondary schools  Adolescent girls  (M=17.1 years,  SD=1.6)  Setting: Secondary schools | 1. Dissonance intervention (D)  2. Expressive writing control intervention (EW)  3. Assessment-only control condition (A) | 3 weeks | Group  3 weekly 1-hour sessions  F2F | Posttest, 6 months, 1 year  2 and 3 years | Primary outcomes | Primary outcomes |
|  |  |  |  |  |  |  | D vs A  ↓Thin-ideal internalization  ↓Body dissatisfaction**  ↓Dieting  ↓Negative affect**  ↓Bulimic symptoms    D vs EW  ↓Thin-ideal internalization*  ↓Body dissatisfaction**  ↓Dieting  ↓Negative affect**  ↓Bulimic symptoms**    *only at posttest  **only at posttest and 6 months  ***only at 6 months and 1 year  ****only at posttest and 1 year  D vs A  ↓Thin-ideal internalization^  ↓Body dissatisfaction  ↓Negative affect  ↓Bulimic symptoms^  ↓Psychosocial impairment   D vs EW  ↓Thin-ideal internalization^  ↓Body dissatisfaction  =Negative affect  =Bulimic symptoms  ↓Psychosocial impairment^^    ^only at 2 years  ^^only at 3 years | D vs A  ↓Obesity    D vs EW  ↓Obesity  D vs A  =BMI    D vs EW  =BMI   ^only at 2 years  ^^only at 3 years |
|  |  |  |  |  |  |  | Secondary outcomes | Secondary outcomes |
|  |  |  |  |  |  |  | =Social functioning |  |
| 15 | Stice et al. (2006b)  USA  (International Journal of Eating Disorders) | College women  (M=21.3 years,   SD=not reported,  Range=19-40 years)  Setting: Colleges/universities | 1. Psychoeducational eating disturbance intervention  2. Psychology seminar (matched comparison group) | 15 weeks | Group  Twice weekly 1.5-hour sessions  F2F | Posttest, 6 months | Primary outcomes | Primary outcomes |
|  |  |  |  |  |  |  | ↓Thin-Ideal internalization  ↓Body dissatisfaction  ↓Dieting  =Depressive symptoms  ↓Eating pathology  =Fat consumption | ↓BMI |
|  |  |  |  |  |  |  | Secondary outcomes | Secondary outcomes |
|  |  |  |  |  |  |  |  |  |
| 16 | Stice et al. (2011)*  USA | Female high school students  (M=15.7 years,  SD=1.1)  Setting: Secondary schools | 1. Dissonance intervention  2. Educational brochure control condition | 4 weeks | Group  Weekly 1-hour session  F2F | 2 and 3 years | Primary outcomes | Primary outcomes |
|  |  |  |  |  |  |  | ↓Body dissatisfaction  =Thin-Ideal internalization  =Dieting  =Depressive symptoms  ↓Eating pathology  ↓Functional impairment  =Health care utilization | =BMI |
|  |  |  |  |  |  |  | Secondary outcomes | Secondary outcomes |
|  |  |  |  |  |  |  |  |  |
| 17 | Stice et al. (2013c)  USA  (Behaviour Research and Therapy) | Female college students with body image concerns  (M=21.6years,  SD=5.64)  Setting: Colleges/universities | 1. The Body Project  2. Educational brochure control condition | 4 weeks | Group  Weekly 1-hour session  F2F | Posttest, 1 year | Primary outcomes | Primary outcomes |
|  |  |  |  |  |  |  | ↓Thin-ideal internalization  ↓Body dissatisfaction  ↓Dieting  ↓Negative affect  ↓Eating disorder symptoms |  |
|  |  |  |  |  |  |  | Secondary outcomes | Secondary outcomes |
|  |  |  |  |  |  |  | ↓Psychosocial functioning  =Health and mental health care utilization | =BMI |
| 18 | Stice at al. (2014)  USA | Female college students with body image concern (M=21.6, SD=6.6)  Setting: Colleges/universities | 1. eBody Project  2. Body Project  3. Video control  4. Brochure control | eBody: 3 weeks  Body: 4 weeks | eBody: six 30-40 mins online module  Body: four 1hr weekly group session of 7-9 participants  (F2F)  Video: 55 min documentary  Brochure: 2page brochure sent through mail. | Posttest, 1 and 2 year | Primary outcomes | Primary outcomes |
|  |  |  |  |  |  |  | eBody Project vs Brochure Controls  ꓿Thin-ideal internalization  ꓿Body dissatisfaction  ꓿Dieting  ꓿Depressive affect  ꓿Eating Disorder Symptoms   eBody Project vs Video Controls  ꓿Thin-ideal internalization  ꓿Body dissatisfaction  ↓Dieting  ꓿Depressive affect  ꓿Eating Disorder Symptoms    eBody Project vs Body Project  ꓿Thin-ideal internalization  ꓿Body dissatisfaction  ꓿Dieting  ꓿Depressive affect  ꓿Eating Disorder Symptoms  Body Project vs Brochure Controls  ꓿Thin-ideal internalization  ꓿Body dissatisfaction  ꓿Dieting  ꓿Depressive affect  ꓿Eating Disorder Symptoms    Body Project vs Video Controls  ꓿Thin-ideal internalization  ↓Body dissatisfaction  ↓Dieting  ꓿Depressive affect  Eating Disorder Symptoms | eBody Project vs Brochure Controls  ↓BMI    eBody Project vs Video Controls  ꓿BMI     eBody Project vs Body Project  ꓿BMI    Body Project vs Brochure Controls  ↑BMI    Body Project vs Video Controls  ꓿BMI |
|  |  |  |  |  |  |  | Secondary outcomes | Secondary outcomes |
|  |  |  |  |  |  |  |  |  |
| 19 | Stice et al. (2015a)  USA  (Behaviour Research and Therapy journal) | Female undergraduate and graduate students, university staff (M= 21.6, SD=5.64); with reported body image concerns; did not meet criteria for DSM-IV at pretest  Setting: Colleges/universities | 1. Body Project  2. Control | 4 weeks | Body Project: 4 weekly 1hr group session with 5-9 participants  (F2F)  Control: 2 educational brochures | Posttest, 1, 2 and 3 years | Primary outcomes | Primary outcomes |
|  |  |  |  |  |  |  | ↓Thin-ideal internalization  ↓Body dissatisfaction  ↓Dieting  ↓Negative affect  ↓Eating Disorder Symptoms  ꓿Eating Disorder onset | ꓿BMI |
|  |  |  |  |  |  |  | Secondary outcomes | Secondary outcomes |
|  |  |  |  |  |  |  | ↓Psychosocial impairment | ꓿BMI |
| 20 | Taylor et al. (2006)  USA | College women  (M=20.8 years,   SD=2.6)  Setting: Colleges/universities | 1. Student Bodies  2. Control | 8 weeks | Individual and group  Weekly cognitive behavioural program combined with an online, asynchronous, moderated discussion group at participant’s convenience  Online | Posttest, 1, 2 and 3 years*    *2 and 3 years only for those recruited in waves 1 and 2 | Primary outcomes | Primary outcomes |
|  |  |  |  |  |  |  | =Clinical/sub-clinical EDs  ↓Weight Concerns Scale  ↓Global Eating Disorder Examination   ↓EDI drive for thinness  ↓EDI bulimia subscale score  =Depression | =BMI |
|  |  |  |  |  |  |  | Secondary outcomes | Secondary outcomes |
|  |  |  |  |  |  |  |  |  |
| 21 | Wilksch et al. (2015)* Australia    *only results for the ED-focused interventions in this study are presented here | Grades 7 and 8 school children  (M=13.21 years,  SD=0.68)  Setting: Secondary schools | 1. Media smart  2. HELPP  3. Control group | 4 weeks | Group  8 lessons of 50-min duration delivered at the rate of two lessons per week  F2F | Posttest, 6 and 12 months | Primary outcomes | Primary outcomes |
|  |  |  |  |  |  |  | Media Smart vs Control:  Weight concern  =girls     =boys    Shape concern   = girls     =boys    Eating concern  =girls    Dieting  =girls    =boys    Body dissatisfaction  =girls    ↓ boys*    Media internalisation  =girls    ↓ boys     Perceived pressure  =girls   =boys    Depression  =girls    ↓ boys***    Weight-related peer teasing  =girls   =boys    Perfectionism  =girls    ↓ boys*    Regular eating  =girls     =boys    Screen time  =girls    =boys      HELPP vs Control:  Weight concern  =girls     =boys    Shape concern  =girls     =boys    Eating concern  ↑girls**    Dieting  = girls    =boys    Body dissatisfaction  =girls      =boys    Media internalisation  = girls    ↓boys****    Perceived pressure  ↑girls**     =boys    Depression  =girls    =boys    Weight-related peer teasing  =girls   =boys    Perfectionism  =girls     =boys    Regular eating  =girls     =boys    Screen time  =girls     =boys    *only at posttest  **only at 6 months  ***only at 12 months  ****only at posttest and 6 months | Media Smart vs Control:  Physical activity  ↑ girls**     =boys    BMI  =girls =boys    HELPP vs Control:  Physical activity  =girls    ↓ boys***    BMI  =girls  =boys    *only at posttest  **only at 6 months  ***only at 12 months  ****only at posttest and 6 months |
|  |  |  |  |  |  |  | Secondary outcomes | Secondary outcomes |
|  |  |  |  |  |  |  |  |  |
| 22 | Zabinski et al. (2001)  USA | College students  (M=24 years,  SD=1.95)  Setting: Colleges/universities | 1. GRAD group (physical activity-based intervention)  2. Control group | 15 weeks | Group  Weekly 1-hr lecture and a1½-hr small group behavior change lab over 15 weeks  F2F | Posttest | Primary outcomes | Primary outcomes |
|  |  |  |  |  |  |  | Drive for thinness  ↑ female     = male    Female concerns/Body dissatisfaction  = Female      = male    Male concerns/body dissatisfaction  ↓female      =male |  |
|  |  |  |  |  |  |  | Secondary outcomes | Secondary outcomes |
|  |  |  |  |  |  |  |  |  |
| 54 | González et al. (2011)  Spain | Adolescents  (M=13.5, SD=0.4)  Setting: Secondary schools | 1. Media Literacy (ML)  2. Media LIteracy + Nutrition Awareness (ML+NUT)  3. Control | 5 weeks | Group  NUT: one 90-minute sessions  ML: two 90-minute sessions plus two 60-minute activity sessions, one session per week for 4 weeks.  NUT+ML: one session per week for 5 weeks  F2F/Class | Posttest, 7 and 30 months | Primary outcomes | Primary outcomes |
|  |  |  |  |  |  |  | ML vs NT  ↓ Eating Attitudes Test (EAT-40)   ↓Influences on body shape model-26 (CIMEC-26)    ML+NUT vs NT  ↓ Eating Attitudes Test (EAT-40)  ↓ Influences on body shape model-26 (CIMEC-26) | ML vs NT  =BMI    ML+NUT vs NT  =BMI |
|  |  |  |  |  |  |  | Secondary outcomes | Secondary outcomes |
|  |  |  |  |  |  |  |  |  |
| **Primary Aim: High BMI prevention** | | | | | | | | |
| 23 | Austin et al. (2005)  USA | Girls grade 6 and 7 aged 10-14 (M=11.5yrs, SD= 0.7)  Setting: Secondary schools | 1. Planet Health  2. Control | 2 school years | 16 (45 minute) classroom lessons for each year | 21 months | Primary outcomes | Primary outcomes |
|  |  |  |  |  |  |  | ↓Purging or use of diet pills | only baseline % of Obese participants were reported |
|  |  |  |  |  |  |  | Secondary outcomes | Secondary outcomes |
|  |  |  |  |  |  |  |  |  |
| 24 | Austin et al. (2007)  USA | Girls and Boys in Grades 6 and 7; without disordered weight-control behaviors at BL.  Setting: Secondary schools | 1. The 5-2-1 Go! (Planet Health curriculum + School Health Index)  2. Control | 2 school years | Planet Health: 16 (45 minute) classroom lessons for each year   SHI: online self-evaluation and planning tool for schools | 18 months | Primary outcomes | Primary outcomes |
|  |  |  |  |  |  |  | disordered weight-control behaviors  ↓Girls  ꓿Boys | physical activity and diet were measured but not reported |
|  |  |  |  |  |  |  | Secondary outcomes | Secondary outcomes |
|  |  |  |  |  |  |  |  |  |
| 25 | deMenezes et al. (2015)  Brazil | Women   (M= 57.9 ± 11.7)  Setting: Community | 1. Transtheoretical Model (TTM)-based intervention  2. Control | 6 months | Twenty 70-min workshops limited to a maximum of 15 participants | Posttest | Primary outcomes | Primary outcomes |
|  |  |  |  |  |  |  | ꓿Body satisfaction (satisfied)  ꓿Body satisfaction (dissatisfied)  ꓿Body perception (Thin or very thin)  ↓Body perception (Little fat.fat. too fat) | ↓BMI  ꓿Waist circumference |
|  |  |  |  |  |  |  | Secondary outcomes | Secondary outcomes |
|  |  |  |  |  |  |  |  |  |
| 26 | Elinder et al. (2012)  Sweden | Children in grades 2,4 and 7 aged 6-16 years  Setting: Primary schools | 1. The Stockholm County Implementation Programme in school (SCIP-school)  2. Comparison group | 26 months | School was asked to form a health team; conduct 4 workshops, four training sessions for school health teams; writing of action plans, conduct a school self- assessment (KEY) and parent gatherings. | 2 years | Primary outcomes | Primary outcomes |
|  |  |  |  |  |  |  | ꓿Self-worth (Harter’s Self-Perception Profile for Adolescents)  ꓿Global Self-esteem  ꓿Dieting | ꓿BMI   ꓿Fruit intake  ꓿Vegetable intake  ꓿Physical Activity |
|  |  |  |  |  |  |  | Secondary outcomes | Secondary outcomes |
|  |  |  |  |  |  |  |  |  |
| 27 | Gow et al. (2010)  USA | Healthy 1st year college students, 22 years or younger (M=18.10)  Setting: Colleges/universities | 1. Combined intervention  2. Internet intervention  3. Feedback intervention  4. Control group | 6 weeks | Internet: six weekly 45 min online, group discussion  Feedback: 6-weekly reporting of self-weight and caloric  feedback afterwards (via email) | Posttest and 3 months*    *analysis of 3mos FU not conducted due to low completion of FU measure | Primary outcomes | Primary outcomes |
|  |  |  |  |  |  |  |  | Combined vs Control:  ↓BMI    Combined vs. Internet:  ↓BMI    Combined vs. Feedback:  ↓BMI    Internet vs. Control:  =BMI    Feedback vs. Control:  =BMI |
|  |  |  |  |  |  |  | Secondary outcomes | Secondary outcomes |
|  |  |  |  |  |  |  | All Interventions*  ꓿Binge Eating Scale (BES)  ꓿Body Rating Scale (BRS)  ꓿EDI - Drive for thinness  ꓿EDI - Body dissatisfaction    Three Factor Eating Questionnaire subscales (TFEQ)  ꓿Restraint  ꓿Disinhibition  ꓿Hunger    *no significant differences on any other measures for all interventions | All Interventions*  ꓿Physical Activity (IPAQ)  ꓿Fruit/Veg intake (Block Food Screener) |
| 28 | Huang et al. (2007)  USA | Adolescents aged 11-15 years  Setting: Community | 1. The Patient-Centered Assessment and Counseling for Exercise Plus Nutrition Project (PACE+) intervention  2. Control group | 1 year | Computer-supported intervention with: computer assessment; 16-section printed manual (Teen Guide) to take home; 12- month stage-matched phone and mail contacts (3-5 mins individual counselling) | 6 and 12 months | Primary outcomes | Primary outcomes |
|  |  |  |  |  |  |  | Body Image  ꓿Girls     ꓿Boys    Self-Esteem  ꓿Girls     ꓿Boys | BMI not reported |
|  |  |  |  |  |  |  | Secondary outcomes | Secondary outcomes |
|  |  |  |  |  |  |  |  |  |
| 29 | Huang et al. (2011)  Taiwan | Pregnant women 18 years or older  Setting: Community | 1. EP (From 16 gestational weeks to six months post-partum)  2. EPP (From birth to six months post-partum)  3. Comparison group | 12 months | Six one-to -one counselling sessions (about 30-40mins) on a regularly  scheduled clinic visits (EP) or delivered at bedside in the obstetric units and  during regularly scheduled clinic visits (EPP). | Posttest | Primary outcomes | Primary outcomes |
|  |  |  |  |  |  |  | EP vs Comparison:  ↑Body Image  ↑Depression (BDI-II)   EPP vs Comparison:  ↑Body Image  ↑Depression (BDI-II)    EP vs. EPP:  ↑Body Image  ↑Depression (BDI-II) | EP vs Comparison:  Body weight changes:  ↓GWG  ↓Weight retention    Health promoting behaviour:  ↑Nutrition  ↑ Physical Activity    EPP vs Comparison:  Body weight changes:  ↓GWG  ↓Weight retention    Health promoting behaviour:  ↑Nutrition  ↑ Physical Activity    EP vs. EPP:  Body weight changes:  ↓GWG  ↓Weight retention    Health promoting behaviour:  =Nutrition  =Physical Activity |
|  |  |  |  |  |  |  | Secondary outcomes | Secondary outcomes |
|  |  |  |  |  |  |  |  |  |
| 30 | Jeffrey et al. (1999)  USA | Male and female aged 20-45 years; not currently pregnant/pregnant in previous year; no serious medical condition requiring treatment  Setting: Community | 1. Education through monthly newsletters (NEWS)  2. Education plus incentives for participation (NEWS+INC)  3. Control group | 3 years | NEWS:2-4 pages monthly newsletter with 5 questions (to be returned) sent through mail; offered every 6mos: face-to-face weight control and physical activity sessions.  INC: $100 lottery held each month after returning newsletter | 1, 2, and 3 year | Primary outcomes | Primary outcomes |
|  |  |  |  |  |  |  | NEWS vs. Control:  =Unhealthy weight loss practices  ↓Health weight loss practices    NEWS+INC vs. Control:  =Unhealthy weight loss practices  =Health weight loss practices    NEWS vs NEWS +INC:  =Unhealthy weight loss practices  =Health weight loss practices | NEWS vs. Control:  ꓿Physical activity  ꓿Weight gain    NEWS +INC vs. Control:  ꓿Physical activity  ꓿Weight gain    NEWS vs NEWS +INC:  ꓿Physical activity  ꓿Weight gain |
|  |  |  |  |  |  |  | Secondary outcomes | Secondary outcomes |
|  |  |  |  |  |  |  |  |  |
| 31 | Katterman et al. (2016)  USA | University women 18-25 years; BMI 20–30 kg/m2; no current or past ED diagnosis  Setting: Colleges/universities | 1. Intervention (daily weight monitoring)  2. Control | 8 weeks | Daily weight monitoring, submitted online | 8 weeks, only weight change was measured at 20 weeks | Primary outcomes | Primary outcomes |
|  |  |  |  |  |  |  | ꓿Body Satisfaction (BBS)  ꓿Depression  ꓿Anxiety  ꓿Unhealthy weight control | ꓿Weight change |
|  |  |  |  |  |  |  | Secondary outcomes | Secondary outcomes |
|  |  |  |  |  |  |  |  |  |
| 32 | Levine et al. (2007)  USA | Healthy women 25-44 years (M=35.6, SD=5.7); with BMI between 21 and 30; not pregnant; did not participated in weight loss program in the past year; no psychiatric disorder; not taking medications affecting body weight.  Setting: Community | 1. Clinic group  2.Correspondence group  3. Information only control | 2 years | Behavioral interventions of: Clinic group: 15 group meetings over a 24-month period (F2F);  Correspondence group: 15 lessons by mail over a 24-month | Posttest, 1, 2 and 3 years | Primary outcomes | Primary outcomes |
|  |  |  |  |  |  |  | Three-factor Eating Questionnaire (TFEQ) subscale:  ↑Restraint at 2 year FU  Disinhibition*  Hunger*   Depression (CES-D)*   Correspondence Group vs Control:  Three-factor Eating Questionnaire (TFEQ) subscale:  Restraint*  Disinhibition*  Hunger*   Depression (CES-D)*  *only reported baseline data | Clinic Group vs Control:  ꓿BMI  Physical activity (PAQ)*    Correspondence Group vs Control:  =BMI  Physical activity (PAQ)* |
|  |  |  |  |  |  |  | Secondary outcomes | Secondary outcomes |
|  |  |  |  |  |  |  |  |  |
| 33 | Lombard et al. (2009)  Australia | Healthy non pregnant women with at least 1 child in primary school. (M age= 40.4, SD=4.7)  Setting: Community | 1. Self-management lifestyle intervention  2. Control | 1 year | 1. 3 interactive 1hour group sessions (10-30 participants) in the 1st month and a 4th session at 4 months + ongoing support thru phone text for 1 year. (F2F)  2.single 30min group, non-interactive lecture.  (F2F) | 4, 12 months | Primary outcomes | Primary outcomes |
|  |  |  |  |  |  |  | Weight related behaviors*    *only reported baseline data | Waist circumference*  Physical activity (IPAQ)*    *only reported baseline data |
|  |  |  |  |  |  |  | Secondary outcomes | Secondary outcomes |
|  |  |  |  |  |  |  |  |  |
| 34 | Mason et al. (2018)  UK | 18 years or older; BMI 20 or above; non-pregnant/ breastfeeding  Setting: Community | 1. Behavioural intervention  2. Control | 4 weeks | Brief behavioural intervention: daily self-weighing, advice for weight management(10TT) and pictorial information about the physical activity calorie equivalent (PACE) | Posttest (within 4 to 8 weeks) | Primary outcomes | Primary outcomes |
|  |  |  |  |  |  |  |  | ↓Weight change |
|  |  |  |  |  |  |  | Secondary outcomes | Secondary outcomes |
|  |  |  |  |  |  |  | ↑Cognitive Restraint  ꓿Emotional eating  ꓿Uncontrolled eating | ꓿% Body Fat  ꓿Weight gain <0.5 kg (OR) |
| 35 | Robinson et al. (2003)  USA | African-American girls  (M=9.5 years,   SD=0.85)*  Setting: Community  *calculated by combining two arms | 1. After-school dance classes and a family-based intervention  2. Active Control | 3 months | Group and individual  2.5 hours dance class 5 days per week  5 home visits  F2F | Posttest | Primary outcomes | Primary outcomes |
|  |  |  |  |  |  |  | ↓Overconcerns with weight and  body shape   =Body shape dissatisfaction | =BMI  =Waist circumference  =Physical activity  ↓Self-reported media use  ↓Meals eaten with TV |
|  |  |  |  |  |  |  | Secondary outcomes | Secondary outcomes |
|  |  |  |  |  |  |  |  |  |
| 36 | Sahota et al. (2001)  UK | School children  (M=8.4 years,  SD=0.63)  Setting: Primary schools | 1. APPLES (active programme promoting lifestyle education) intervention   2. Control | 12-month academic year | Group  Programme consisted of teacher training, modifications of school meals, and the development and implementation of school action plans designed to promote healthy eating and physical activity over one academic year  F2F | Posttest | Primary outcomes | Primary outcomes |
|  |  |  |  |  |  |  | ↑Global self-worth  =Dietary restraint  =Body perception | ↑Vegetable consumption  =BMI  =Physical activity  ↓Fruit consumption |
|  |  |  |  |  |  |  | Secondary outcomes | Secondary outcomes |
|  |  |  |  |  |  |  |  |  |
| 37 | Sahota et al. (2019)  UK | School children  (M=7.2 years,  SD=1.13)*  Setting: Primary schools  *calculated by combining two arms | 1. PhunkyFoods Programme (PFP)  2. Control | 17 months (2 academic years) | Group  Multi-format school curriculum programme  F2F | 6 and 18 months | Primary outcomes | Primary outcomes |
|  |  |  |  |  |  |  | ↑Healthy lifestyle knowledge  =Diet and lifestyle behaviour  =Body shape satisfaction  =Dieting behaviours | =BMI |
|  |  |  |  |  |  |  | Secondary outcomes | Secondary outcomes |
|  |  |  |  |  |  |  |  |  |
| 38 | Salmon et al. (2008)  Australia | School children   (M=10.67 years,  SD=0.38)  Setting: Primary schools | 1. Behavioural modification (BM)  2. Fundamental movement skills (FMS)  3. Combined BM/FMS  4. Control | 1 school year (9 months) | Group  19 lessons (40–50 min each)  F2F | Posttest, 6 and 12 months | Primary outcomes | Primary outcomes |
|  |  |  |  |  |  |  | BM  =Body image concerns*   FMS  =Body image concerns*    BM/FMS  =Body image concerns*  *Investigated as unintended outcomes | BM  =BMI  =Overweight/obese  ↑Physical activity  ↑Self-reported screen behaviours  =Self-reported enjoyment of physical activity   =Fundamental movement skills    FMS  =BMI  =Overweight/obese  ↑Physical activity  =Self-reported screen behaviours  ↑Self-reported enjoyment of physical activity   =Fundamental movement skills    BM/FMS  ↓BMI  ↓Overweight/obese  =Physical activity  =Self-reported screen behaviours  =Self-reported enjoyment of physical activity   =Fundamental movement skills |
|  |  |  |  |  |  |  | Secondary outcomes | Secondary outcomes |
|  |  |  |  |  |  |  |  |  |
| 11 | Stice et al. (2001)  USA  (A randomized trial of a dissonance-based eating disorder prevention program) | Female young adults  (Age range 17-29 years, mean age and SD not reported)  Setting: Colleges/universities | 1. Dissonance-based intervention  2. Healthy weight management control | 3 weeks | Group  3 weekly 1-hour sessions  F2F | Posttest, 4 weeks | Primary outcomes | Primary outcomes |
|  |  |  |  |  |  |  | ↓Thin-Ideal internalization  ↓Body dissatisfaction  =Dieting  =Negative affect  =Bulimic symptoms  =Fat consumption | =BMI  =Exercise behaviours |
|  |  |  |  |  |  |  | Secondary outcomes | Secondary outcomes |
|  |  |  |  |  |  |  |  |  |
| 13 & 14 | Stice et al. (2006a)  USA  (Journal of Consulting and Clinical Psychology)  &     Stice et al. (2008a)  USA  (Journal of Consulting and Clinical Psychology) | Adolescent girls  (M=17.0 years,  SD=1.4)  Setting: Colleges/universities  Adolescent girls  (M=17.1 years,  SD=1.6) | 1. Healthy weight intervention (HW)  2. Expressive writing control intervention (EW)  3. Assessment-only control condition (A) | 3 weeks | Group  3 weekly 1-hour sessions  F2F | Posttest, 6 months, 1 year  2 and 3 years | Primary outcomes | Primary outcomes |
|  |  |  |  |  |  |  | HW vs A  ↓Thin-ideal internalization  ↓Body dissatisfaction**  ↓Dieting***  ↓Negative affect*  ↓Bulimic symptoms***    HW vs EW  ↓Thin-ideal internalization****  ↓Body dissatisfaction**  =Dieting  ↓Negative affect*  ↓Bulimic symptoms**    *only at posttest  **only at posttest and 6 months  ***only at 6 months and 1 year  ****only at posttest and 1 year  HW vs A  ↓Thin-ideal internalization^^  ↓Body dissatisfaction  ↓Negative affect  ↓Bulimic symptoms  =Psychosocial impairment    HW vs EW  ↓Thin-ideal internalization^^  ↓Body dissatisfaction^  =Negative affect  =Bulimic symptoms  =Psychosocial impairment    ^only at 2 years  ^^only at 3 years | HW vs A  ↓Obesity    HW vs EW  ↓Obesity  HW vs A  ↓BMI^^   HW vs EW  ↓BMI^^   ^only at 2 years  ^^only at 3 years |
|  |  |  |  |  |  |  | Secondary outcomes | Secondary outcomes |
|  |  |  |  |  |  |  | =Social functioning |  |
| 46 & 47 | Stice et al. (2012a)  USA  (J Consult Clin Psychol)    &    Stice et al. (2013a)  USA  (J Consult Clin Psychol) | Female college students  (M=18.4 years,  SD=0.6)  Setting: Colleges/universities | 1. Healthy Weight 2  2. Educational brochure control condition | 4 weeks | Group  Weekly 1-hour session  F2F | Posttest, 6 months, 1 and 2 years | Primary outcomes | Primary outcomes |
|  |  |  |  |  |  |  | ↓Eating Disorder Diagnostic Interview*    *at posttest, 1 and 2 years only | ↓BMI*    *at 6 months only |
|  |  |  |  |  |  |  | Secondary outcomes | Secondary outcomes |
|  |  |  |  |  |  |  | ↓Body dissatisfaction*  ↓Dietary restraint**  =Depressive symptoms  ↓Perceived Sociocultural Pressure Scale**    *at posttest, 1 and 2 years only  **at posttest and 6 months only | ↑Physical activity*  =Dietary intake  *at posttest only |
| 39 | Stice et al. (2015b)  USA  (Physiology & Behavior journal) | Young adults with weight concerns; no current ED diagnosis    (M= 19.3 ± 1.2)  Setting: Colleges/universities | 1. Minding Health  2. Healthy weight  3. Video control | Minding health: 7 weeks;  Healthy weight: 6 weeks | 1.Minding Health: 7 1-h weekly group  meetings  of 6–10 participants  (F2F)  2.Healthy weight: 6 1-h weekly group meetings of 6–10 participants  (F2F)  3.Control: 51min video | Posttest and 6 months | Primary outcomes | Primary outcomes |
|  |  |  |  |  |  |  | Minding Health vs Control  =Eating disorder symptom        Healthy Weight vs Control  =Eating disorder symptom         Healthy Weight vs Minding Health  ↓Eating disorder symptom | Minding Health vs Control  =BMI  =% body fat    Healthy Weight vs Control  =BMI  =% body fat   Healthy Weight vs Minding Health  =BMI  =% body fat |
|  |  |  |  |  |  |  | Secondary outcomes | Secondary outcomes |
|  |  |  |  |  |  |  |  |  |
| 48 | Stice et al. (2018)  USA*    *only results for the high BMI-focused intervention in this study are presented here | College students with weight concerns aged 17-23 (M age = 19.1, SD = 1.2); BMI between 18 and 30; no ED diagnosis  Setting: Colleges/universities | 1. Healthy Weight (HW)  2. Control | 6 weeks | HW and PH: 6 weekly 1hr group session with 6-10 participants  (F2F)    Control:51 min video | Posttest, 6, 12, 24 months | Primary outcomes | Primary outcomes |
|  |  |  |  |  |  |  | HW vs Control:  ↓Eating disorder symptoms  ꓿Cognitive dissonance | HW vs Control:  =BMI |
|  |  |  |  |  |  |  | Secondary outcomes | Secondary outcomes |
|  |  |  |  |  |  |  |  |  |
| 40 | Timmerman & Brown (2012)  USA | Healthy, perimenopausal women who eat out frequently  (M=49.6 years,  SD=6.8)  Setting: Community | 1. Mindful Restaurant Eating  2. Control | 6 weeks | Group  Weekly 2-hour session  F2F | Posttest | Primary outcomes | Primary outcomes |
|  |  |  |  |  |  |  | =Emotional eating  ↑Self-efficacy for eating behaviors  ↓Barriers to weight management in restaurant eating | ↓Weight  ↓Dietary intake  =Waist circumference |
|  |  |  |  |  |  |  | Secondary outcomes | Secondary outcomes |
|  |  |  |  |  |  |  |  |  |
| 41 | West et al. (2016)  USA | College students  (M= 21.6 years,  SD=2.2)  Setting: Colleges/universities | 1. Healthy weight (behavioural weight gain prevention intervention)  2. Control group | 9 weeks | Individual and group  8 technology-mediated sessions  Online | Posttest | Primary outcomes | Primary outcomes |
|  |  |  |  |  |  |  | ↑Appropriate weight control strategies  =Inappropriate weight control strategies | =BMI |
|  |  |  |  |  |  |  | Secondary outcomes | Secondary outcomes |
|  |  |  |  |  |  |  |  |  |
| **Primary Aim: Eating Disorder (ED) and High BMI prevention** | | | | | | | | |
| 42 | Castillo et al. (2019)  Mexico | Male and female students aged 17-28 (M=19.78, SD=2.06);  BMI ≥ 17.5 kg/m2 or ≤30.0 kg/m2  Setting: Colleges/universities | 1. Experimental group Obesity and Eating Disorders (EG_OBEyTA)  2. Control Group Study Skills (CG_SS)  3. Non‐ Intervention Group (CG_NI) | 4 weeks | Eight 90-min group (15-39 participants) workshop conducted 2 days a week after regular classes  F2F | Posttest, & 3-months | Primary outcomes | Primary outcomes |
|  |  |  |  |  |  |  | Sociocultural Attitudes Towards Appearance Questionnaire‐3(SATAQ‐3)  ꓿Male    ↓Female    Disordered Eating Attitudes (EAT‐26)  ꓿Male   ↓Female |  |
|  |  |  |  |  |  |  | Secondary outcomes | Secondary outcomes |
|  |  |  |  |  |  |  | Body dissatisfaction  ꓿Male    ꓿Female    Self‐esteem (RSE)  ꓿Male   ꓿ Female    Self‐perfectionism  ꓿Male    ꓿Female | BMI  ↑Male   ꓿Female    Physical Activity (IPAQ)  ꓿Male    ꓿Female |
| 43 | Dunker et al. (2018)  Brazil | Girls 12-14 years  (M=13.4 years)  Setting: Secondary schools | 1. New Moves program (NMP)  2. Control group | Two non-consecutive blocks of nine weeks | Program content: "Be active"-two  one-hour group sessions weekly for 9 weeks   "Be Fueled" and "Be Fabulous" -one weekly group session lasting  one hour for eight weeks  Two sessions of individual counselling.  F2F | 18 weeks | Primary outcomes | Primary outcomes |
|  |  |  |  |  |  |  | ꓿Body Shape Questionnaire |  |
|  |  |  |  |  |  |  | Secondary outcomes | Secondary outcomes |
|  |  |  |  |  |  |  | ꓿Rosenberg Self-Esteem Scale  ꓿Unhealthy Weight-Control Behaviours | ꓿BMI |
| 44 | Leme et al. (2019)  Brazil | 1st to 3rd year high school girls (M=15.6 years, SD=0.05); not enrolled in any health-related course  Setting: Secondary schools | 1. Healthy Habits, Healthy Girls-Brazil (H3G-Brazil)  2. Control | 6 months | 10 key-messages with activities delivered twice a week (3hr/week), delivered by teachers, WhatsApp weekly messages, diaries and parents' newsletter. | Posttest and 6 months | Primary outcomes | Primary outcomes |
|  |  |  |  |  |  |  | ꓿Body satisfaction  ꓿Healthy weight control behaviors  ↑Unhealthy weight control behaviors  ꓿Extreme unhealthy weight control behaviors  ꓿Other weight control behaviors  ꓿Binge eating | ꓿Physical activity  ↑Health eating strategies (FV intake etc.) |
|  |  |  |  |  |  |  | Secondary outcomes | Secondary outcomes |
|  |  |  |  |  |  |  |  |  |
| 45 | Neumark-Sztainer et al. (2010)  USA | Inactive girls  (M=15.8 years, SD=1.17)  Setting: Secondary schools | 1. New Moves  2. Control wait list | 9-month school year | Individual and group  16 weeks PE sessions  5-7 individual counselling sessions  F2F | Posttest, 9 months | Primary outcomes | Primary outcomes |
|  |  |  |  |  |  |  | ↓Unhealthy weight control behaviours  ↑Body/self-image | ꓿Percent body fat  ꓿BMI  ↑Physical activity  ↓Sedentary activity  ↑Dietary intake  ↑Eating patterns |
|  |  |  |  |  |  |  | Secondary outcomes | Secondary outcomes |
|  |  |  |  |  |  |  |  |  |
| 48 | Stice et al. (2018)  USA | College students with weight concerns aged 17-23 (M age = 19.1 years, SD = 1.2); BMI between 18 and 30; no ED diagnosis  Setting: Colleges/universities | 1. Project Health (PH)  2. Control | 6 weeks | HW and PH: 6 weekly 1hr group session with 6-10 participants  (F2F)    Control:51 min video | Posttest, 6, 12, 24 months | Primary outcomes | Primary outcomes |
|  |  |  |  |  |  |  | PH vs Control:  ↓Eating disorder symptoms  ↑Cognitive dissonance | PH vs Control:  ↑BMI |
|  |  |  |  |  |  |  | Secondary outcomes | Secondary outcomes |
|  |  |  |  |  |  |  |  |  |
| 49 | Stock et al. (2007)  Canada | Elementary school children  (Mean age and SD not reported)  Kindergarten through 7th grade  Setting: Primary schools | 1. Healthy Buddies  2. Control | 21 weeks | Individual and group (peer system)  2-3 hours per week  F2F | Posttest | Primary outcomes | Primary outcomes |
|  |  |  |  |  |  |  | ↑Healthy Living Questionnaire  =Harter Self-competence Scales  =Body-Image Perception  =Children’s Eating Attitude Test | ↓BMI*  =Fitness    *Only in the 4th through 7th grade group |
|  |  |  |  |  |  |  | Secondary outcomes | Secondary outcomes |
|  |  |  |  |  |  |  |  |  |
| 50 | Tanofsky-Kraff et al. (2010)  USA | High school girls  (M=15.05 years,  SD=0.92)*  Setting: Secondary schools  *Calculated by combining mean and SD of two groups | 1. Interpersonal psychotherapy weight gain (IPT)-WG  2. Standard health education (HE) | 12 weeks | Group  Weekly session  F2F | 6 months, 1 year | Primary outcomes | Primary outcomes |
|  |  |  |  |  |  |  | =Number of group sessions attended and completion of follow-up visits |  |
|  |  |  |  |  |  |  | Secondary outcomes | Secondary outcomes |
|  |  |  |  |  |  |  | ↓Loss of control eating | ↓BMI |
| 51 & 52 | Tanofsky-Kraff et al. (2014)  USA  &    Tanofsky-Kraff et al. (2017)  USA | Girls 12-17years  (M = 14.50 years, SD = 1.65);  BMI between 75th and 97th percentile; LOC episodes  Setting: Secondary schools | 1. Interpersonal psychotherapy group (IPT)  2. Health education group (HE) | 12 weeks | 90-min pre-group  individual meeting with each girl, followed by 12 consecutive, weekly 90-min group sessions  F2F | Posttest, 6-month, 1 and 3 years | Primary outcomes | Primary outcomes |
|  |  |  |  |  |  |  | ↓LOC-eating status  ↓Depression  ↓Anxiety    There were no ED results reported for 3 years | ↓BMIz*  ↓Body Fat Mass (%)*    *only at posttest, 6 months and 1 year |
|  |  |  |  |  |  |  | Secondary outcomes | Secondary outcomes |
|  |  |  |  |  |  |  |  |  |
| 53 | Wilksch et al. (2013) Australia | Grade 7 girls and boys  (M=12.71, SD=0.41)  Setting: Secondary schools | 1. Life Smart  2. Control group | 4 weeks | Group  School-based curriculum. 8-lesson Life Smart program at the rate of two 50-min lessons per week.  F2F | Posttest | Primary outcomes | Primary outcomes |
|  |  |  |  |  |  |  | Dieting  =girls     =boys    Body dissatisfaction  ↓girls      = boys    Depression  =girls       =boys    Media internalisation  =girls      = boys    Concern over mistakes  =girls     =boys    Peer teasing  ↓girls       = boys    Eating habits  = girls       = boys    Screen time  = girls       = boys    Shape and weight concern  ↓girls      =boys | Physical activity  = girls      = boys |
|  |  |  |  |  |  |  | Secondary outcomes | Secondary outcomes |
|  |  |  |  |  |  |  |  |  |
| 21 | Wilksch et al. (2015)* Australia    *only results for the ED and high BMI-focused intervention in this study are presented here | Grades 7 and 8 school children  (M=13.21 years,  SD=0.68)  Setting: Secondary schools | 1. Life Smart  2. Control group | 4 weeks | Group  8 lessons of 50-min duration delivered at the rate of two lessons per week  F2F | Posttest, 6 and 12 months | Primary outcomes | Primary outcomes |
|  |  |  |  |  |  |  | Life Smart vs Control:  Weight concern  =girls     =boys    Shape concern  =girls     =boys    Eating concern  ↑ girls***    Dieting  =girls     =boys    Body dissatisfaction  =girls      ↓ boys*    Media internalisation  =girls    =boys    Perceived pressure  =girls    =boys    Depression  =girls    =boys    Weight-related peer teasing  =girls   =boys     Perfectionism  =girls     =boys    Regular eating  =girls     =boys    Screen time  =girls    =boys    *only at posttest  **only at 6 months  ***only at 12 months  ****only at posttest and 6 months | Life Smart vs Control:   Physical activity  =girls =boys    BMI  =girls =boys    *only at posttest  **only at 6 months  ***only at 12 months  ****only at posttest and 6 months |
|  |  |  |  |  |  |  | Secondary outcomes | Secondary outcomes |
|  |  |  |  |  |  |  |  |  |

**Table S2b**. Risk of bias for included studies.

| **Risk of bias summary** | | | | | | | |
| --- | --- | --- | --- | --- | --- | --- | --- |
|  | Random sequence generation (selection bias) | Allocation concealment (selection bias) | Blinding of participants and personnel (performance bias) | Blinding of outcome assessors for all outcomes (detection bias) | Incomplete outcome data (attrition bias) | Selective outcome reporting (reporting bias) | Other sources of bias |
| Armitage 2015 |  |  | **** | **** | **** | **** | **** |
| Austin 2005 |  |  |  |  | **** | **** |  |
| Austin 2007 |  |  |  |  | **** | **** | **** |
| Bearman 2003 |  |  |  |  | **** | **** | **** |
| Castillo 2019 | **** | **** |  |  | **** | **** | **** |
| deMenezes 2015 | **** | **** |  | **** | **** | **** | **** |
| Dunker 2018 | **** | **** | **** | **** | **** | **** | **** |
| Elinder 2012 | **** | **** |  |  | **** | **** | **** |
| Gow 2010 | **** | **** |  |  | **** | **** | **** |
| Gonzalez 2011 |  | **** |  |  | **** | **** | **** |
| Huang 2007 |  |  |  |  | **** | **** | **** |
| Huang 2011 | **** | **** | **** | **** | **** | **** | **** |
| Jacobi 2007 | **** | **** |  |  | **** | **** | **** |
| Jacobi 2012 | **** |  |  | **** | **** | **** | **** |
| Jeffery 1999 |  |  |  |  | **** | **** | **** |
| Kass 2014 | **** | **** |  |  | **** | **** | **** |
| Katterman 2016 | **** | **** |  |  | **** | **** | **** |
| Leme 2019 | **** | **** |  | **** | **** | **** | **** |
| Levine 2007 |  |  |  |  | **** | **** | **** |
| Lombard 2009 | **** | **** | **** | **** | **** |  | **** |
| Lopez-Guimera 2011 | **** |  | **** |  |  | **** | **** |
| Mason 2018 | **** | **** | **** | **** | **** | **** | **** |
| Neumark-Sztainer 2010 |  |  |  | **** | **** | **** | **** |
| O'Brien 2007 |  |  |  | **** | **** | **** | **** |
| Olive 2019 | **** | **** | **** | **** | **** | **** | **** |
| Plessow 2018 | **** | **** | **** | **** | **** | **** | **** |
| Robinson 2003 | **** | **** | **** | **** | **** | **** |  |
| Sahota 2001 | **** |  | **** | **** |  | **** | **** |
| Sahota 2019 | **** | **** | **** | **** |  | **** | **** |
| Salmon 2008 | **** |  | **** | **** | **** | **** | **** |
| Sanchez-Carracedo 2016 | **** | **** |  | **** | **** | **** | **** |
| Stice 2001 |  |  |  | **** | **** | **** | **** |
| Stice 2002 | **** | **** |  |  | **** | **** | **** |
| Stice 2006a | **** |  |  | **** | **** | **** | **** |
| Stice 2006b | **** | **** |  | **** | **** | **** | **** |
| Stice 2008a |  |  | **** | **** | **** |  | **** |
| Stice 2011 | **** |  | **** | **** | **** | **** | **** |
| Stice 2012 | **** |  |  | **** | **** | **** | **** |
| Stice 2013a | **** |  |  |  | **** | **** | **** |
| Stice 2013c | **** | **** |  | **** | **** | **** | **** |
| Stice 2014 | **** | **** | **** | **** | **** | **** | **** |
| Stice 2015a | **** | **** |  | **** | **** | **** | **** |
| Stice 2015b |  |  |  | **** | **** | **** | **** |
| Stice 2018 | **** | **** |  | **** | **** | **** | **** |
| Stock 2007 | **** | **** |  | **** |  | **** | **** |
| Tanofsky-Kraff 2010 | **** | **** |  | **** | **** | **** | **** |
| Tanofsky-Kraff 2014 | **** | **** |  | **** | **** | **** | **** |
| Tanofsky-Kraff 2017 | **** | **** |  |  | **** | **** | **** |
| Taylor 2006 | **** | **** |  | **** | **** | **** | **** |
| Timmerman 2012 | **** | **** | **** | **** | **** | **** | **** |
| West 2016 | **** |  | **** |  | **** | **** | **** |
| Wilksch 2013 | **** | **** |  |  | **** | **** | **** |
| Wilksch 2015 | **** |  |  | **** | **** | **** | **** |
| Zabinski 2001 |  |  |  |  |  | **** | **** |

** **Low risk of bias  Unclear risk of bias ** **High risk of bias

**Table S3a. Summary of pooled effect results of the preventive interventions of eating disorders and high BMI**

| **Outcome Title** | **No. of studies at post-test (at follow up)** | **No. of participants at post-test (at follow up)** | **Statistical method** | **Pooled effect at post-test [95% CI]**  **(Q, p, I^2^)** | **Pooled effect at up to 1 year follow-up [95% CI]**  **(Q, p, I^2^)** | **Measure Scale** |
| --- | --- | --- | --- | --- | --- | --- |
| **Dieting** | | | | | | |
| All measures | 15 (13)       (5) | 2631 (2218)       (1219) | SMD (Hedges’g, QE, 95% CI)    WMD (WMD, QE, 95% CI) | **-0.24 [-0.32, -0.16]**  (Q=24.05, p=0.19, I^2^=21%) | **-0.24 [-0.31, -0.17]**  (Q=15.99, p=0.52, I^2^=0%)    *-0.14 [-0.28, 0.00]  (Q=10.70, p=0.10, I^2^=44%) | EDE-Q Restraint, DRES, DEBQ, DIET    DRES |
| **Shape and Weight Concern** | | | | | | |
| All measures | 6 (5) | 1346 (1259) | SMD (Hedges’g, QE, 95% CI) | **-0.42 [-0.69, -0.15]**  (Q=42.57, p=0.00, I^2^=84%) | **-0.45 [-0.63, -0.27]**  (Q=15.29, p=0.02, I^2^=61%) | McKnight RF, WCS, CIMEC |
| Weight Concern Scale (WCS) | 3 (3) | 590 (590) | WMD (WMD, QE, 95% CI) | -9.37 [-19.19, 0.46]  (Q=17.08, p=0.00, I^2^=88%) | -5.92 [-9.33, -2.52]  (Q=2.67, p=0.26, I^2^=25%) | WCS |
| EDE-Q Weight Concern | 2 (2) | 199 (199) | WMD (WMD, QE, 95% CI) | -0.20 [-0.49, 0.08]  (Q=0.00, p=1.00, I^2^=0%) | -0.30 [-0.58, -0.02]  (Q=1.04, p=0.31, I^2^=4%) | EDE-Q Weight Concern |
| EDE-Q Shape Concern | 2 (2) | 199 (199) | WMD (WMD, QE, 95% CI) | -0.10 [-0.41, 0.21]  (Q=0.00, p=NAN, I^2^=0%) | -0.29 [-0.58, 0.00]  (Q=0.88, p=0.35, I^2^=0%) | EDE-Q Shape Concern |
| CIMEC | 2 (2) | 695 (669) | WMD (WMD, QE, 95% CI) | -4.33 [-6.29, -2.37]  (Q=8.44, p=0.04, I^2^=64%) | -5.69 [-8.18, -3.21]  (Q=10.84, p=0.01, I^2^=72%) | CIMEC |
| **Body Dissatisfaction** | | | | | | |
| All measures | 21 (16) | 4827 (3597) | SMD (Hedges’g, QE, 95% CI) | **-0.21 [-0.30, -0.12]**  (Q=68.93, p=0.00, I^2^=59%) | -**0.17 [-0.24, -0.09]**  (Q=27.16, p=0.17, I^2^=23%) | SDBPS, McKnight RF – body dissatisfaction, EDI-BD, EDI2-BD, EDI3-BD, MBAS, BSQ, NM, BSES-C, ABIS, DBI |
| Satisfaction and Dissatisfaction with Body Parts Scale (SDBPS) | 11 (9)      (6) | 2021 (1523)      (1700) | WMD (WMD, QE, 95% CI)  WMD (WMD, QE, 95% CI) | -0.32 [-0.43, -0.21]  (Q=25.75, p=0.03, I^2^=46%) | -0.20 [-0.28, -0.12]  (Q=10.76, p=0.55, I^2^=0%)    *-0.12 [-0.21, -0.02]  (Q=16.82, p=0.08, I^2^=41%) | SDBPS  SDBPS |
| Eating Disorder Inventory-2 (EDI-2): Body Dissatisfaction | 2 (3) | 199 (856) | WMD (WMD, QE, 95% CI) | -0.11 [-0.47, 0.26]  (Q=0.07, p=0.79, I^2^=0%) | -0.31 [-0.80, 0.18]  (Q=2.26, p=0.32, I^2^=12%) | EDI-2 BD |
| **Negative Affect** | | | | | | |
| All measures | 14 (12)        (5) | 2932 (2606)        (1394) | SMD (Hedges’g, QE, 95% CI)      SMD (Hedges’g, QE, 95% CI) | **-0.25 [-0.33, -0.19]**  (Q=18.51, p=0.42, I^2^=3%) | **-0.13 [-0.22, -0.04]**  (Q=20.41, p=0.16, I^2^=27%)      *-0.05 [-0.14, 0.04]  (Q=5.02, p=0.83, I^2^=0%) | PANAS-X Sadness, PANAS-N, BDI, SADSSC, CESD    PANAS-X Sadness, BDI, SADSSC |
| **Eating Disorder Symptoms** | | | | | | |
| All measures | 22 (19)  (9) | 4834 (4303)  (2756) | SMD (Hedges’g, QE, 95% CI)  SMD (Hedges’g, QE, 95% CI) | **-0.18 [-0.25, -0.12]**  (Q=40.29, p=0.08, I^2^=28%) | **-0.16 [-0.27, -0.06]**  (Q=37.63, p=0.07, I^2^=31%)  ***-0.61 [-0.29, -0.04]**  (Q=42.14, p=0.00, I^2^=67%) | EDE-Q Bulimic Symptoms, EDI Bulimia, EDDI, EDDS, Global EDE-Q, ChEAT, EAT-26, EES, TFEQ, EDE-Q Eating Concern  EDE-Q Bulimic Symptoms, EDDI, EAT-26 |
| Eating Disorder Examination–Questionnaire (EDE-Q): Bulimic symptoms | 3 (3) | 641 (641) | WMD (WMD, QE, 95% CI) | -0.12 [-0.24, 0.01]  (Q=12.25, p=0.03, I^2^=59%) | -0.10 [-0.18, -0.02]  (Q=6.90, p=0.23, I^2^=28%) | EDE-Q BS |
| Eating Disorder Inventory (EDI): Bulimia | 2 (2) | 493 (493) | WMD (WMD, QE, 95% CI) | -0.03 [-0.74, 0.68]  (Q=6.59, p=0.01, I^2^=85%) | -0.15 [-0.36, 0.07]  (Q=0.56, p=0.45, I^2^=0%) | EDI B |
| Eating Disorder Diagnostic Interview (EDDI) | 8 (7)      (6) | 1731 (1425)      (1583) | WMD (WMD, QE, 95% CI)    WMD (WMD, QE, 95% CI) | -1.22 [-1.82, -0.63]  (Q=8.10, p=0.62, I^2^=0%) | -0.67 [-1.40, 0.06]  (Q=6.96, p=0.64, I^2^=0%)    *-1.02 [-1.68, -0.37]  (Q=3.09, p=0.93, I^2^=0%) | EDDI  EDDI |
| Eating Disorder Diagnostic Scale (EDDS) | 2 (1) | 161 (95) | WMD (WMD, QE, 95% CI) | -0.76 [-5.94, 4.42]  (Q=1.11, p=0.29, I^2^=10%) | 1.30 [-5.23, 7.85]  (Q=0.00, p=NAN, I^2^=0%) | EDDS |
| Eating Attitudes Test-26 (EAT-26) | 3 (3) | 886 (799) | WMD (WMD, QE, 95% CI) | -2.24 [-4.86, 0.38]  (Q=22.12, p=0.00, I^2^=82%) | -2.74 [-5.71, 0.22]  (Q=22.20, p=0.00, I^2^=82%) | EAT-26 |
| Eating Disorder Examination-Questionnaire (EDE-Q): Eating Concern (EDE-Q EC) | 2 (2) | 199 (199) | WMD (WMD, QE, 95% CI) | 0 [-0.13, 0.13]  (Q=0.00, p=n/a, I^2^=0%) | -0.04 [-0.22, 0.13]  (Q=1.25, p=0.26, I^2^=20%) | EDE-Q Eating Concern |
| **Anthropometric** | | | | | | |
| All measures | 18 (15)  (8) | 2835 (2852)        (2274) | SMD (Hedges’g, QE, 95% CI)  SMD (Hedges’g, QE, 95% CI) | 0.02 [-0.05, 0.09]  (Q=20.18, p=0.45, I^2^=1%) | -0.03 [-0.11, 0.05]  (Q=23.34, p=0.22, I^2^=19%)  *-0.04 [-0.14, 0.05]  (Q=20.42, p=0.12, I^2^=31%) | BMI, Weight change, Percent body fat, Waist circumference, Age-adjusted percentile    BMI, Weight change, Age-adjusted percentile |
| BMI | 16 (15)  (6) | 2539 (2790)  (1758) | WMD (WMD, QE, 95% CI)  WMD (WMD, QE, 95% CI) | 0.01 [-0.24, 0.27]  (Q=18.01, p=0.46, I^2^=0%) | -0.13 [-0.45, 0.18]  (Q=24.52, p=0.10, I^2^=31%)  *-0.33 [-0.77, 0.11]  (Q=15.54, p=0.16, I^2^=29%) | BMI  BMI |
| Percent body fat | 2 | 409 | WMD (WMD, QE, 95% CI) | -0.21 [-1.86, 1.44]  (Q=0.53, p=0.77, I^2^=0%) |  | Percent body fat |
| Waist circumference | 2 | 106 | WMD (WMD, QE, 95% CI) | 1.21 [-7.91, 10.33]  (Q=3.86, p=0.05, I^2^=74%) |  | Waist circumference |
| **Physical Activity** | | | | | | |
| All measures | 5 (3) | 916 (611) | SMD (Hedges’g, QE, 95% CI) | 0.21 [-0.10, 0.53]  (Q=23.11, p=0.00, I^2^=78%) | -0.13 [-0.29, 0.03]  (Q=2.60, p=0.63, I^2^=0%) | Accelerometer, IPAQ, HPLP, PYLPAS, PAQ |
| **Unhealthy Behaviour** | | | | | | |
| All measures | 3 | 311 | SMD (Hedges’g, QE, 95% CI) | -0.15 [-0.53, 0.23]  (Q=5.21, p=0.07, I^2^=62%) |  | BWCP, SCI-DSM IV, EDE |
| **Internalization** | | | | | | |
| All measures | 10 (8) | 2279 (1781) | SMD (Hedges’g, QE, 95% CI) | **-0.42 [-0.58, -0.27]**  (Q=43.03, p=0.00, I^2^=67%) | **-0.25 [-0.34, -0.15]**  (Q=14.39, p=0.28, I^2^=17%) | SATAQ-3 Internalization General, IBSS thin-ideal internalization |
| Sociocultural attitudes toward appearance questionnaire - 3 (SATAQ-3): Internalization General (IG) | 2 (2) | 729 (603) | WMD (WMD, QE, 95% CI) | -0.22 [-2.14, 1.71]  (Q=1.02, p=0.31, I^2^=2%) | -1.83 [-4.13, 0.48]  (Q=1.13, p=0.29, I^2^=12%) | SATAQ-3 Internalization General |
| Ideal-Body Stereotype Scale-Revised (IBSSR) (thin-ideal internalization) | 8 (6)      (5) | 1550 (1178)      (1302) | WMD (WMD, QE, 95% CI)    WMD (WMD, QE, 95% CI) | -0.34 [-0.40, -0.29]  (Q=9.50, p=0.58, I^2^=0%) | -0.18 [-0.26, -0.10]  (Q=14.41, p=0.16, I^2^=31%)    *-0.12 [-0.18, -0.05]  (Q=12.95, p=0.16, I^2^=31%) | IBSS thin-ideal internalization |
| **Drive for Thinness** | | | | | | |
| All measures | 5 (4) | 1446 (1053) | SMD (Hedges’g, QE, 95% CI) | -0.27 [-0.59, 0.05]  (Q=27.11, p=0.00, I^2^=85%) | **-0.30 [-0.49, -0.11]**  (Q=5.61, p=0.13, I^2^=47%) | EDI3-DT, EDI-DT, EDI2-DT |
| EDI DT | 2 | 710 | WMD (WMD, QE, 95% CI) | -1.93 [-5.94, 2.06]  (Q=24.10, p=0.00, I^2^=96%) |  | EDI-DT |
| EDI-2 DT | 2 (2) | 199 (199) | WMD (WMD, QE, 95% CI) | -0.26 [-2.93, 2.42]  (Q=3.66, p=0.06, I^2^=73%) | -0.47 [-3.90, 2.95]  (Q=5.26, p=0.02, I^2^=81%) | EDI2-DT |
| **Self-esteem** | | | | | | |
| All measures | 2 (3) | 729 (1260) | WMD (WMD, QE, 95% CI) | 0.33 [-0.61, 1.27]  (Q=0.29, p=0.59, I^2^=0%) | 0.34 [-0.45, 1.13]  (Q=2.66, p=0.26, I^2^=25%) | RSES |
| SMD: Standardized Mean Difference; WMD: Weighted Mean Difference; QE: Quality Effect Model; EDE-Q: Eating Disorder Examination – Questionnaire; DRES: Dutch Restrained Eating Scale; DEBQ: Dutch Eating Behavior Questionnaire; DIET: Diet and Preoccupation with Food. McKnight RF: McKnight Risk Factor Survey - overconcerns with weight and shape subscale; WSC: Weight and Shape Concern; CIMEC: Questionnaire on influences of aesthetic body ideal. SDBPS: Satisfaction and Dissatisfaction with Body Parts Scale; EDI-BD: Eating Disorder Inventory – Body Dissatisfaction scale; MBAS: Male Body Attitudes Scale; BSQ: Body Satisfaction Questionnaire; NM: New Moves study; BSES-C: Body Self Esteem Scale for Children; ABIS: Attitude towards Body Image Scale; DBI: Distress about Body Image. PANAS-X: the Positive Affect and Negative Affect Scale-Revised; BDI: Beck Depression Inventory; SADSSC: Schedule for Affective Disorders and Schizophrenia for School-age Children; CESD: The Center for Epidemiologic Studies – Depression Scale. EDI: Eating Disorder Inventory; EDDI: The Eating Disorder Diagnostic Interview; EDDS: The Eating Disorder Diagnostic Survey. BMI: Body Mass Index; IPAQ: International Physical Activity Questionnaire; HPLP: Health Promoting Lifestyle Profile; PYLPAS: Past Year Leisure Physical Activity Scale; PAQ: Paffenbarger Activity Questionnaire. BWCP: Behavioral Weight Control Practices checklist; SCI-DSM IV: Structured Clinical Interview for DSM IV Axis1 Disorders: ED Diagnostic Section; EDE: Eating Disorder Examination - episodes in the past 28 days. SATAQ: Sociocultural Attitudes Towards Appearance Questionnaire; IBSS: The Ideal-Body Stereotype Scale; EDI-DT: Eating Disorder Inventory – Drive for Thinness scale. RSES: Rosenberg Self-Esteem Scale. ChEAT: Eating Attitudes Test – Children version; EAT-26: Eating Attitudes Test-26; EES: Emotional Eating Scale; TFEQ: The Three Factor Eating Questionnaire.  *At second follow up: >1 year of follow up.  Figures in parentheses are at follow up.  Q = Cochran’s Q test statistic  p = p-value for Q  I^2^ = I squared test statistic | | | | | | |

**Table S3b. Risk of publication bias: Results of LFX index**

| **Outcome** | **LFK index at**  **post test**  **(Risk of**  **publication bias)** | **LFK index at**  **follow up, 0-1 year**  **(Risk of**  **publication bias)** | **LFK index at**  **follow up, >1 year**  **(Risk of**  **publication bias)** |
| --- | --- | --- | --- |
| Dieting | -0.53 (No) | -1.11 (Minor) | -6.04 (Major) |
| Shape and weight concern | 2.61 (Major) | 1.36 (Minor) | n/a |
| Body dissatisfaction | -1.67 (Minor) | -2.60 (Major) | -1.03 (Minor) |
| Negative affect | 0.19 (No) | -0.75 (No) | 0.71 (No) |
| Eating disorder symptoms | 0.92 (No) | 0.30 (No) | -0.13 (No) |
| Anthropometric | 0.02 (No) | -1.65 (Minor) | -0.43 (No) |
| Physical activity | 0.05 (No) | -1.07 (Minor) | n/a |
| Unhealthy behaviour | 2.33 (Major) | n/a | n/a |
| Internalization | -0.04 (No) | -0.54 (No) | 2.58 (Major) |
| Drive for Thinness | -1.37 (Minor) | -0.90 (No) | n/a |
| Self-esteem | n/a | -0.56 (No) | n/a |

Note: LFK index of asymmetry which was developed by Luis Furuya-Kanamori assess the severity (‘No if LFK index within ±1’, ‘Minor if LFK index exceeds ±1 but within ±2’, and ‘Major’ if LFK index exceeds ±2) of risk of publication bias.
